# Redescription of *Antetintinnidium mucicola* (Claparède and Lachmann, 1858) nov. gen., nov. comb. (Alveolata, Ciliophora, Tintinnina)

**DOI:** 10.1111/jeu.12728

**Published:** 2019-03-12

**Authors:** Maximilian H. Ganser, Sabine Agatha

**Affiliations:** Department of Biosciences, University of Salzburg, 5020 Salzburg, Austria

**Keywords:** Biogeography, cladistic analysis, marine plankton, morphology, phylogeny, somatic ciliary pattern, tintinnid

## Abstract

Tintinnid ciliates have traditionally been described and classified exclusively based on their lorica features. Although information on the cell characters is urgently needed for a natural classification, more molecular than cytological data has been accumulated over recent years. Apparently, the tintinnids developed in the marine environment and entered freshwater several times independently. Typical freshwater tintinnids belong to the genera *Tintinnidium* and *Membranicola*. The species are comparatively well-known regarding their morphology and characterised by two unusual de novo originating ciliary rows, the ventral organelles. In contrast, the cell features in the marine/brackish *Tintinnidium* species, specifically their somatic ciliary patterns, are insufficiently known or not known at all. Therefore, the morphology of a common marine/brackish representative, *Tintinnidium mucicola*, is redescribed based on live observation and protargol-stained material. Furthermore, biogeographical and autecological data of the species are compiled from literature and own records. The phylogenetic relationships of *T. mucicola* are inferred and the diversity of the family Tintinnidiidae is assessed from 18S rDNA sequences. The study shows that *T. mucicola* is not only molecularly distinct, but also characterised by many plesiomorphic features, for instance, it does not possess a verifiable homologue to the ventral organelles. Hence, a new genus, *Antetintinnidium* nov. gen., is established for *T. mucicola*. The new insights into the diversity of Tintinnidiidae shed light on the early evolution of tintinnids and might provide clues on their adaptions to freshwater.

TINTINNID ciliates and their ability to form a wide variety of houses, called loricae, were already recognised in the 19th century (Claparède and Lachmann 1858). Further studies led to the description of more than 1,000 species based on lorica morphology, mostly compiled in two comprehensive monographs by Kofoid and Campbell (1929, 1939). Although these books still serve as references for the current lorica-centred taxonomy of tintinnids, it is highly recommended to use original descriptions or authoritative redescriptions for species identification. The reliable identification, using lorica morphology, is indispensable to link the few species redescriptions lacking gene sequences and the many gene sequences missing descriptions of the cytological features with a species name and with each other. An approach providing all data from the same population is optimal (Santoferrara et al. 2016b). Assembling the main pillars (live observation, protargol-staining, gene sequencing, and ecological data) from different populations spatially and temporarily apart is suboptimal and only possible if conspecificity is beyond reasonable doubt.

The monophyly of the Tintinnina Kofoid and Campbell, 1929 (Spirotricha, Choreotrichida) is supported by molecular and morphological data (Agatha and Strüder-Kypke 2007; Santoferrara et al. 2012; Strüder-Kypke and Lynn 2003). However, some tintinnid families and, especially genera turn out to be not monophyletic in phylogenies (Bachy et al. 2012) mainly because of homoplasious lorica features. For example, species building robust houses with agglutinated particles, but without a collar, have historically been assigned to the genus *Tintinnopsis* Stein, 1867. Yet, molecular genealogies show that these species are, in fact, scattered over several distinct clades and closely related to tintinnid taxa with different lorica structures (Santoferrara et al. 2017). Using the lorica as the sole character to create a tintinnid classification has already been criticised very early on in several studies (Brandt 1907; Bütschli 1887; Hofker 1931). Despite technological advances in microscopy, features of the tintinnid cells are still known in merely a small fraction of the named species, which is insufficient for the urgently required revision of the tintinnid systematics (Agatha and Strüder-Kypke 2014).

Tintinnids are suitable models for studies on the ecology, diversity, and biogeography of microbial plankton (Echevarria et al. 2014; Montagnes 2013; Santoferrara et al. 2016a, 2018). They exhibit biogeographic distribution patterns and different tintinnid communities can be found in coastal compared to oceanic regions (Pierce and Turner 1993). Estuaries are especially interesting coastal systems because marine, brackish, and freshwater habitats merge into each other and are therefore characterised by salinity gradients (Telesh and Khlebovich 2010). The diversity of tintinnids in general, and specifically in these transition zones, might be higher than previously known, as suggested by the recent discovery of a new genus (Smith et al. 2018). Some of the species might be rare and/or occur in low abundances and are thus not easily found in samples analysed microscopically. One example is the genus *Nolaclusilis* Snyder and Brownlee, 1991. Its two species have not been barcoded as yet, but might have already been detected by environmental sequencing (Santoferrara et al. 2018).

One of the few tintinnid families known to include marine/brackish as well as freshwater species is the family Tintinnidiidae Kofoid and Campbell, 1929. It currently comprises the genera *Tintinnidium* Saville-Kent, 1881 and *Membranicola* Foissner, Berger, and Schaumburg, 1999 (Santoferrara et al. 2017). The affiliation of the genus *Leprotintinnus* Jörgensen, 1900 with this family changed due to recent molecular data, placing it in a different clade as sister to *Tintinnopsis radix* (Zhang et al. 2017). This is supported by preliminary observations reporting a somatic ciliary pattern similar to the complex ones of other marine tintinnids with a ventral kinety (S. Agatha, unpubl. data), while the somatic ciliary patterns of the tintinnidiid genera *Tintinnidium* and *Membranicola* remained simple.

Congruently, the sparse cytological data and the more comprehensive gene trees indicate a basal branching of the Tintinnidiidae within the tintinnids, forming a sister group to the other families (Agatha and Strüder-Kypke 2014; Bachy et al. 2012; Santoferrara et al. 2012; StrüderKypke and Lynn 2008). Actually, the type genus *Tintinnidium* Saville-Kent, 1881 with its characteristic gelatinous and thus soft lorica was regarded as primitive by Kofoid and Campbell (1939). The investigation of the somatic ciliary patterns in freshwater tintinnidiids revealed unique ciliary structures, the ventral organelles, characterised by a de novo origin during ontogenesis (Foissner and Wilbert 1979; Petz and Foissner 1993). These ventral organelles are regarded as a strong synapomorphy of the Tintinnidiidae (Agatha and Strüder-Kypke 2012; Petz and Foissner 1993). So far, only the lorica morphology of marine/brackish *Tintinnidium* species is known (Barría de Cao 1981; Busch 1923; Claparède and Lachmann 1858), except for a few illustrations showing the ciliary pattern of protargolstained *T. mucicola* specimens (Laval-Peuto 1994; Small and Lynn 1985).

Recent molecular phylogenies place some of the marine/brackish species in clades together with freshwater congeners, while other clades comprise exclusively marine/brackish species (Santoferrara et al. 2017; Zhang et al. 2017). The phylogenetic position of the frequently recorded marine/brackish species *Tintinnidium mucicola* (Claparède and Lachmann, 1858) von Daday, 1887 suggests that it might possess plesiomorphic features providing clues pivotal for understanding the early tintinnid evolution (Agatha and Strüder-Kypke 2013), specifically for inferring the characteristics of the tintinnid ancestor. The small line drawings of *T. mucicola* provided by Small and Lynn (1985) and Laval-Peuto (1994) give merely an impression of the cytological features, but they are accompanied neither by descriptions nor by morphometric data. The depicted specimens apparently differ from their congeners in the absence of ventral organelles. The available morphological data are insufficient for establishing a new genus (Agatha and Strüder-Kypke 2007), although the genetic data and illustrations indicate that *T. mucicola* is distinct from its comparatively well-studied freshwater congeners. Therefore, its lorica and cell morphology are described here based on live observation and protargol-stained material. Furthermore, the cell division pattern is analysed with special emphasis on the development of the ventral somatic ciliature. These morphological and ontogenetic data are included in cladistic analyses and compared with a phylogeny of 18S rDNA sequences retrieved from the NCBI GenBank.

## Materials and Methods

### Collection

The samples were taken in the Chesapeake Bay, Maryland, USA (37°44′N, 76°11′W), in August 1990 by means of vertical net tows (35 μm mesh size) in surface waters (0–10 m). The water temperatures were 20–21 °C and the salinities 14–16‰. This material was used for morphometric analyses after protargol staining. Live observation was conducted on further samples collected in Annapolis Harbour (38°58′37″N, 76°29′04″W), Chesapeake Bay, in May 2009 at water temperatures of 15–18 °C and a salinity of 10‰. Additional live observations are from North Sea specimens studied in August 2014. These samples were taken at the Mariensieler sluice in the Ems-Jade Canal (53°30′49″N, 08°03′11″E) at a water temperature of 18 °C and a salinity of 22‰.

### Taxonomic studies

Cell morphology was investigated under compound microscopes equipped with high-power oil immersion objectives, utilising bright-field and interference contrast optics. The samples taken in 1990 were preserved in a modified Bouin’s fixative (Coats and Heinbokel 1982) and stained by employing the Quantitative Protargol Stain method (Montagnes and Lynn 1987). Counts and measurements on protargol-stained cells were performed at 1,250x magnification with a Leitz Diaplan microscope equipped with a Leica DFC420 digital camera. In vivo measurements were conducted at 250–1,000x magnification.

Protargol-stained slides with the neotypes of *Tintinnidium pusillum* (Inv. No. 1993/64 > MP93_49, 1993/65 > MP93_50) and *Tintinnidium semiciliatum* (Inv. No. 1993/37 > MP93_22, 1993/38 > MP93_23) kindly provided by Dr. Erna Aescht from the Biology Centre of the Museum of Upper Austria (LI) in Linz, Austria, were used for comparison (more than ten specimens of each species were studied from the slides), particularly regarding the ventral organelles. The ventral organelles were additionally recorded in a live *Tintinnidium* specimen (Fig. 9C) collected from Lake Mondsee, Austria. Unfortunately, a neotype of the type species *Tintinnidium fluviatile* was apparently not deposited.

### Illustrations

The drawing of the live specimen is based on free-hand sketches and mean measurements combining the available information. The line drawings of the protargolstained specimens were made by means of a drawing device. The kinetal map shows the ciliary pattern of a morphostatic specimen in two dimensions (Foissner and Wilbert 1979) and is based on mean measurements of protargol-stained morphostatic cells or early dividers. In the latter case, only features that are not influenced by cell division were considered, e.g., the number of somatic ciliary rows. The features of the ciliary pattern are schematised: (i) horizontal bars represent the polykinetids of the collar membranelles, while diagonal bars represent the elongated collar membranelles and the buccal membranelle; (ii) taxonomically relevant curvatures of kineties are considered, viz., the oblique orientation of the first row and the anterior curvature of the last kinety; and (iii) the somatic cilia are shown as perpendicular lines originating from the respective basal bodies, not regarding their individual lengths. Some micrographs are composed of stacked images from several focal planes, utilising the software Picolay (www.picolay.de).

### Terminology

The terminology follows Agatha and Riedel-Lorjé (2006).

### Cladistic analyses

The phylogenetic relationships of *Antetintinnidium mucicola* nov. gen., nov. comb. (basionym *Tintinnus mucicola*) and the freshwater *Tintinnidium* species were analysed, using the computer programs Winclada ver. 1.00.08 (Nixon 2002) for editing the data matrix as well as TNT ver. 1.5 (Goloboff and Catalano 2016) for computing the parsimony trees.

The data matrix established by Agatha and Strüder-Kypke (2014) was extended by a further character, namely, the habitat. Since the majority of extant tintinnids, aloricate choreotrichids, and oligotrichids are marine compared to only a few freshwater species, the most parsimonious assumption is that freshwater was colonised several times independently. Accordingly, ‘marine/brackish’ represents the plesiomorphic character state (coded 0) and ‘freshwater’ the apomorphic state (coded 1). Correspondingly, single species representing terminal taxa were categorised related to their occurrence, while genera representing terminal taxa were categorised based on the most frequently occurring character state (Wiens 2000). Furthermore, *A. mucicola* nov. gen., nov. comb. was added and its characters were coded based on the morphological data acquired in the present study. The new morphological data and additional reinvestigations also necessitated changes in the matrix concerning the presence of a right and left ciliary field in *T. fluviatile*, *T. pusillum*, *Membranicola tamari*, and *Tintinnopsis cylindrata*.

The data matrix was subsequently analysed with TNT, utilising the ‘traditional search’ option and equal weighting of all character states (the ordered/unordered optimisations were adopted from Agatha and Strüder-Kypke 2014). The most parsimonious trees were calculated, using the following parameters: memory set to hold 100,000 trees; Wagner optimisation with starting tree = 1 and 10,000 replicates, tree bisection reconnection (TBR) algorithm saving 10 trees per replication and zero-length branches collapsed. Subsequently, a standard bootstrap resampling with 1,000 replicates was conducted. Additionally, Bremer support values were calculated (Bremer 1994), using the same settings while retaining the suboptimal trees up to two steps longer than the most parsimonious ones. Both the bootstrap and the Bremer support values were mapped on a strict consensus tree. For comparison, a 50% majority-rule consensus tree was calculated from the same tree dataset. Both trees were plotted with Figtree v. 1.4.3 (http://tree.bio.ed.ac.uk/software/figtree/).

### Phylogenetic analyses

Gene sequences were neither obtained from specimens collected in 1990 during a monitoring focusing on quantitative and qualitative aspects nor in 2009 when the species was rare.

The 18S rDNA sequences of 198 oligotrichid, choreotrichid and outgroup taxa (*Oxytricha longa*, *Stylonychia lemnae*, *Halteria grandinella*) were retrieved from NCBI GenBank based on the curation by Santoferrara et al. (2017). The sequences were aligned with MAFFT v. 7 (Katoh and Standley 2013). Ambiguous positions were identified and deleted with Gblocks v. 0.91b (Castresana 2000), using the default parameters, except for allowing gap positions, resulting in a final alignment 1,672 positions long.

A further dataset consisting of all available ‘*Tintinnidium’* sequences as well as similar environmental sequences (≥ 94% identity to tintinnidiid sequences) was retrieved from the NCBI nucleotide database (last updated in August 2018), using the BLASTN algorithm (Zhang et al. 2000). These sequences were then added to the existing alignment by applying the ‘add’ function in MAFFT v. 7 (Katoh and Frith 2012), while keeping the alignment length and using the fast progressive method (Katoh et al. 2002). Duplicates and sequences not clustering with *Tintinnidium* taxa were identified by calculating a distance tree with the neighbour-joining algorithm (Saitou and Nei 1987) in MEGA v. 7 (Kumar et al. 2016) and subsequently removed from the alignment. A maximum likelihood tree was computed from the resulting alignment, including the 198 sequences from the initial dataset and 20 sequences from the second dataset (Table S2) with IQ-TREE (Trifinopoulos et al. 2016). The GTR + Γ + I substitution model was chosen for the tree calculation based on the result of jModelTest v. 2.1.10 (Darriba et al. 2012) under the Akaike Information Criterion. Tree node support was determined from 1,000 bootstraps with the ultrafast bootstrap analysis (Minh et al. 2013), the SH-aLRT branch test (Guindon et al. 2010) with 1,000 replicates, and an approximate Bayes test (Anisimova et al. 2011). Furthermore, the pairwise distances of tintinnidiid sequences were calculated in MEGA v. 7 (Kumar et al. 2016) based on the Kimura-2-Parameter model (Kimura 1980).

## Results

### *Antetintinnidium mucicola* (Claparède and Lachmann, 1858) nov. gen., nov. comb

#### Description of neotype population from the North Atlantic

**Figure 1:**
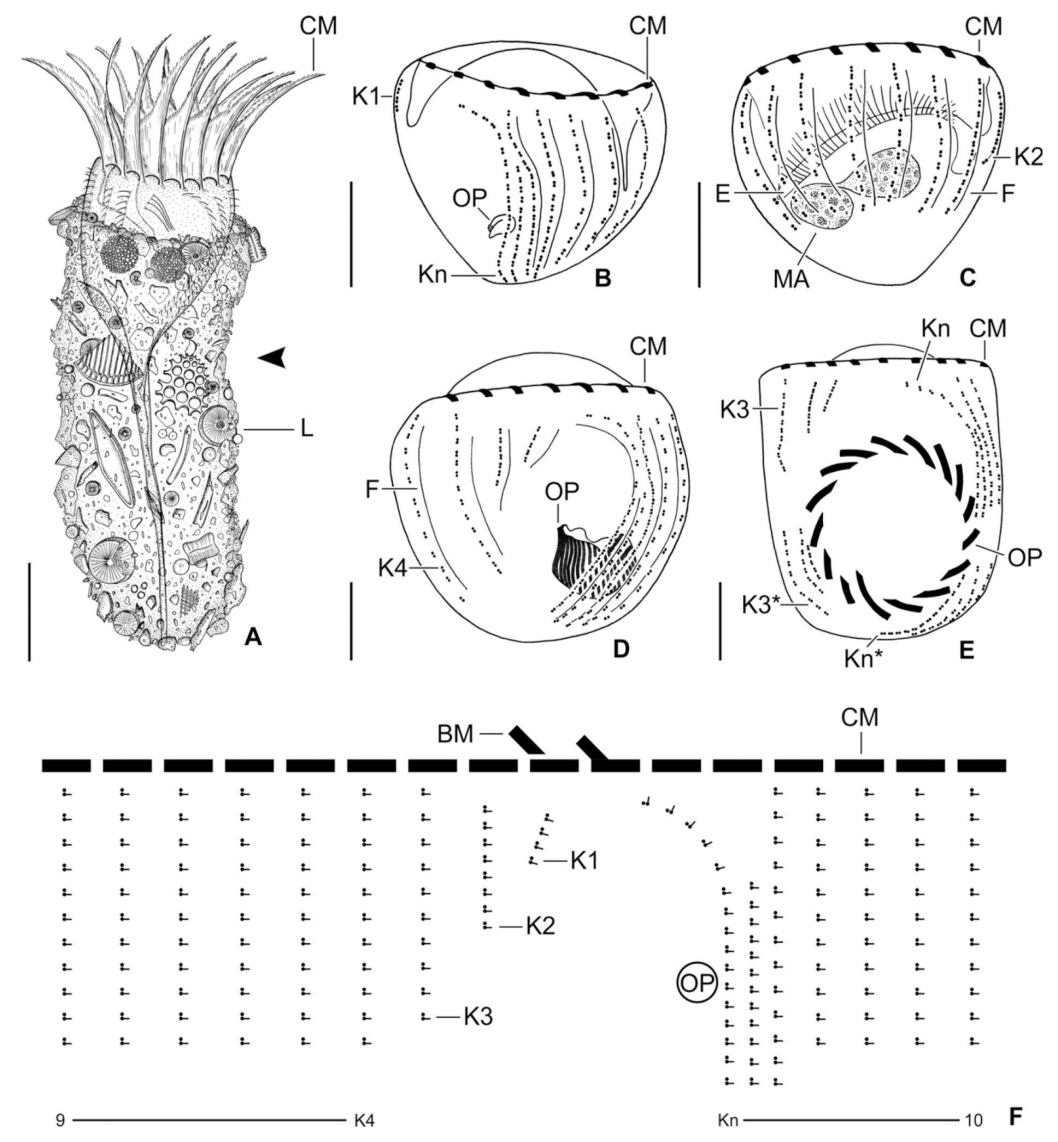
*Antetintinnidium mucicola* nov. gen., nov. comb., North Atlantic specimens from life (A) and after protargol staining (B–F). (**A**) Lateral view of an extended specimen. Note the shallow lateral concavity of the lorica (arrowhead). (**B, C**) Ventrolateral and dorsolateral views of same very early divider depicting the ciliary pattern, the two macronucleus nodules, and the conspicuous endoral membrane. (D, E) Ventral views of an early and a late divider showing the ciliary rows just before proliferation (**D**) and after their split (**E**). (**F**) Kinetal map of a morphostatic specimen. BM = buccal membranelle; CM = collar membranelles; E = endoral membrane; F = argyrophilic fibres; K1–K*n* = kineties 1–*n* of the proter; K3* and Kn* = kineties 3 and *n* of the opisthe; L = lorica; MA = macronucleus nodules; OP = oral primordium. Scale bars = 20 μm (A) and 10 μm (B–E).

Cylindroidal lorica 69–107 × 33–50 μm in size after protargol staining, rarely up to 120 μm long in vivo, slightly asymmetric due to shallow lateral concavity in anterior half, posterior portion broadly rounded (Fig. 1A, 2A). Lorica wall composed of a soft matrix with scarce, but homogenous agglutination of biogenic and abiogenic particles (Fig. 1A, 2B). Outer lorica surface with adhered large diatom frustules and their fragments and detritus clumps containing small diatom frustules; small frustules also embedded in matrix material. Opening rim irregular, without special features.

**Figure 2:**
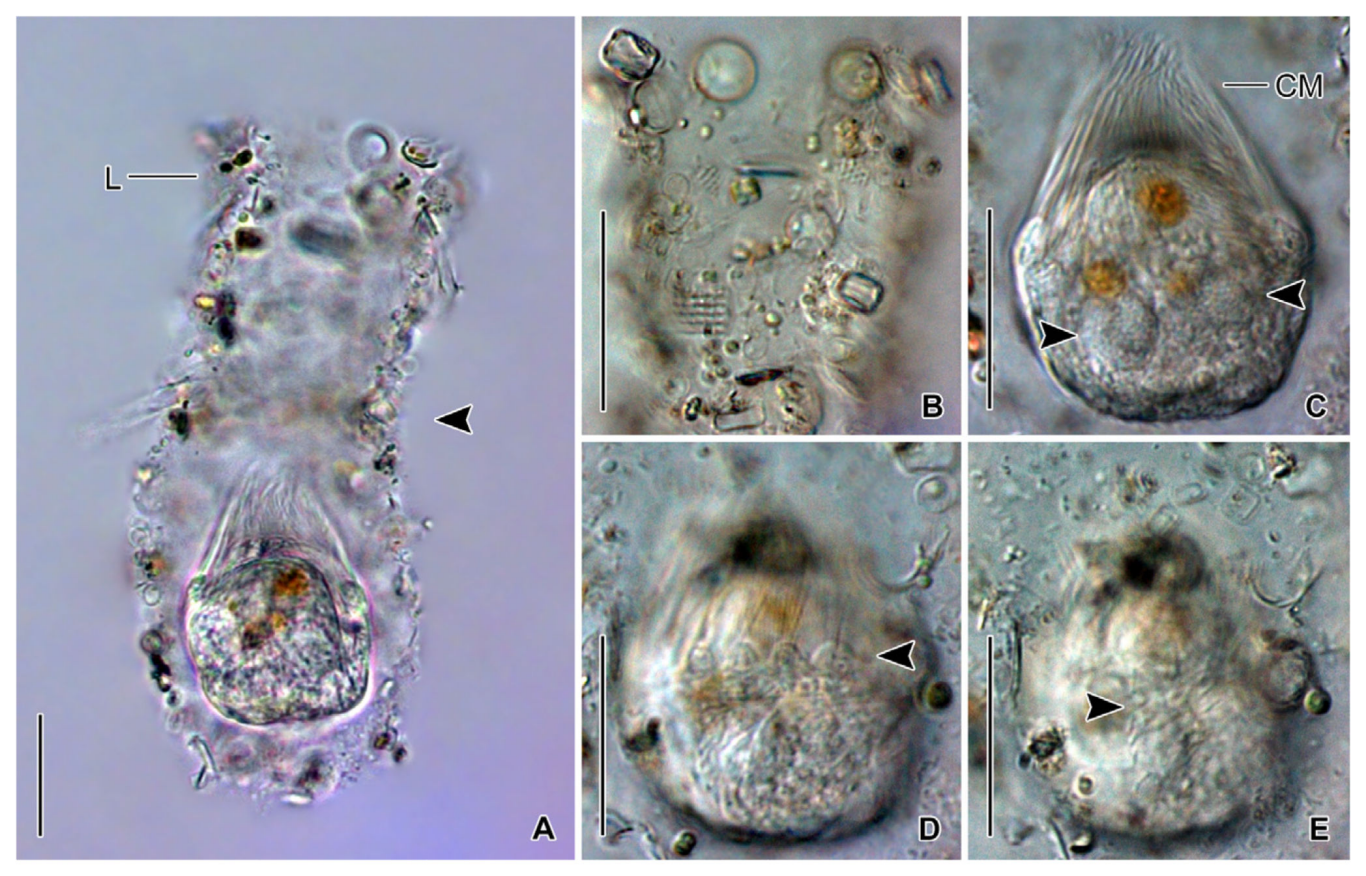
*Antetintinnidium mucicola* nov. gen., nov. comb., live specimens from the North Atlantic. (**A**) Contracted specimen in its lorica. Note the shallow lateral concavity of the lorica (arrowhead). (**B**) Lorica surface with adhered diatom frustules and further particles. (**C**) Specimen showing the two granular macronucleus nodules (arrowheads). (**D**) Specimen showing the distinct ridges between the collar membranelles (arrowhead). (**E**) Somatic kineties on dorsal side (arrowhead). CM = collar membranelles; L = lorica. Scale bars = 20 μm.

Undisturbed living cell obconical, up to 125 × 30 μm in size, attached to the bottom of the lorica by a highly contractile peduncle. During locomotion and feeding, the anterior cell portion extends distinctly beyond the opening rim of the lorica (Fig. 1A). The disturbed cell quickly retracts into the posterior portion of the lorica, becoming subspherical (Fig. 2A, C–E) and about 35 × 30 μm in size, while 20–31 × 23–30 μm after protargol staining; the peduncle is completely retracted and thus not recognisable. Two globular macronucleus nodules (about 8–9 μm across) in the posterior half of the contracted live cell, with finely granulated composition (Fig. 2C). In protargolstained morphostatic specimens, nodules broadly ellipsoidal to globular (5–8 × 5–6 μm in size), usually connected by a thin horizontal isthmus, contain several nucleoli about 1–2 μm across (Fig. 1C, 3B, D). One globular micronucleus about 1 μm across, adjacent to a macronucleus nodule. Distinct intermembranellar ridges (accessory combs) about 3 μm wide, visible in live but not in protargol-stained specimens (Fig. 1A, 2D). Tentaculoids and striae not recognisable. Cytoplasm colourless, with some brownish inclusions, probably remnants of food items (Fig. 2A, C). Contractile vacuole and cytopyge not detectable.

Somatic ciliature composed of usually 16 exclusively dikinetidal kineties, with cilia about 6 μm (*n* = 1) long after protargol staining associated only with the posterior dikinetidal basal bodies, except for the first kinety with motile cilia about 11 μm (*n* = 1) long (Table 1; Fig. 1A–C, F, 3A–D). Ventral side with broad unciliated stripe. Kineties widely and equidistantly spaced, except for densely spaced last three kineties (Fig. 1B, F, 2E, 3A–D). Most kineties extend longitudinally between the membranellar zone and the posterior polar region, on average 15–16 μm long. Kineties 3–14 and 16 comprise usually eight or nine dikinetids per 10 μm, while kinetids more densely spaced in remaining kineties. Four extraordinary kineties on both sides of unciliated ventral stripe: the first, second, penultimate, and last kineties. First kinety markedly clockwise inclined, originates somewhat more posteriorly than the majority of kineties, shortest kinety with invariably 4 μm length, comprises only four or five dikinetids. Second kinety longitudinal, commences at the same level as the first kinety, 6–9 μm long, posteriorly shortened, comprises 7–9 dikinetids. Last kinety commences directly posteriorly to collar membranelles, performs a wide leftwards curvature in its anterior portion and extends with its posterior portion parallel to the penultimate kinety, ending subterminally. Penultimate kinety is longitudinal, distinctly shortened anteriorly, commencing 6–9 μm posteriorly to membranellar zone, ends subterminally at the level of the last kinety.

Unique system of argyrophilic fibres associated with the somatic ciliature. The darkly stained fibres extend parallel to the left side of each kinety (Fig. 1B–D, 3B–D). More lightly stained fibres extend from the posterior dikinetidal basal bodies horizontally leftwards, apparently abutting the longitudinal fibres.

**Figure 3:**
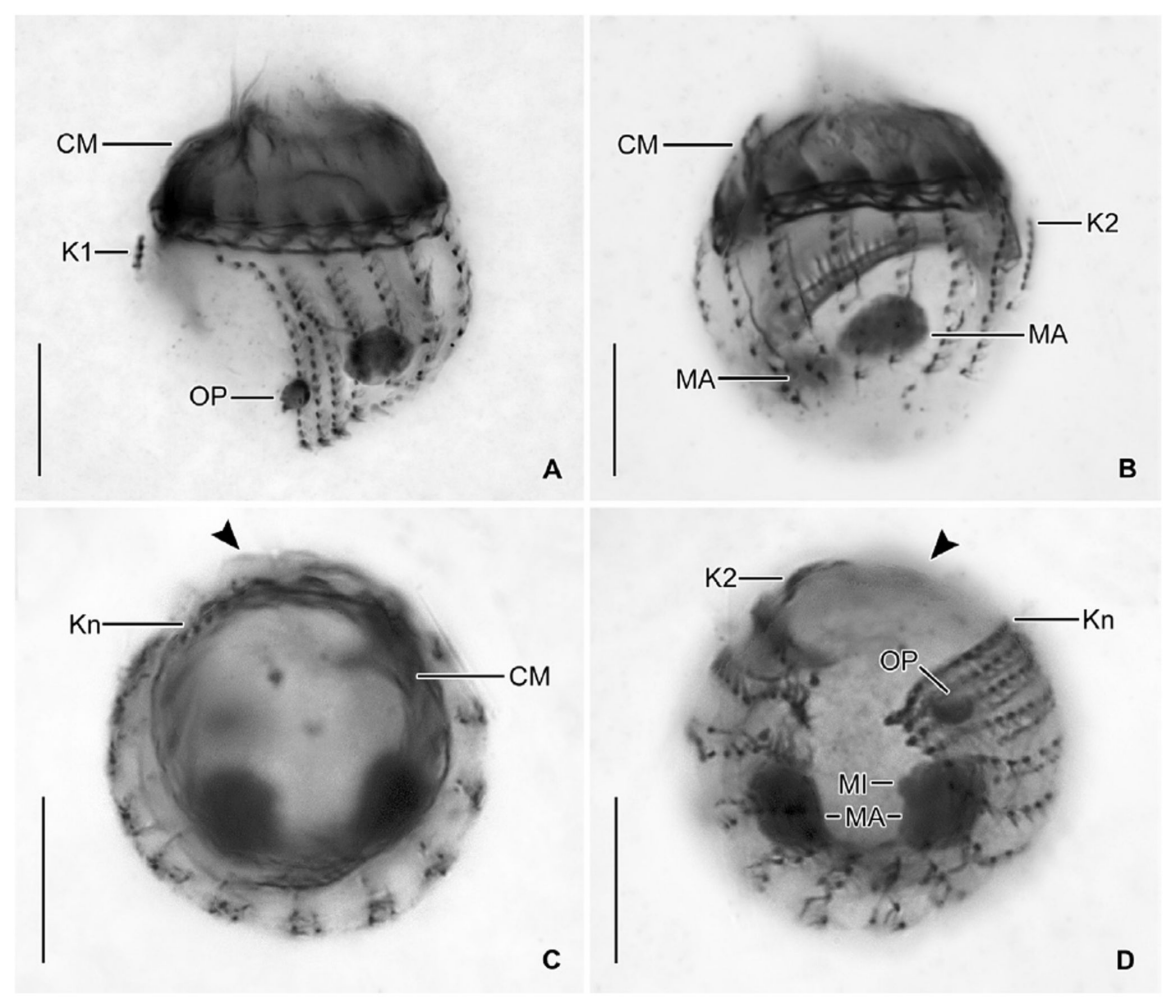
*Antetintinnidium mucicola* nov. gen., nov. comb., North Atlantic specimens after protargol staining. (**A, B**) Ventrolateral and dorsolateral views of type specimen. (**C, D**) Top and posterior polar views of same early divider (stacked images). Arrowheads mark the unciliated ventral stripe. CM = collar membranelles; K1, K2, K*n* = kineties 1, 2, *n*; MA = macronucleus nodules; MI = micronucleus; OP = oral primordium. Scale bars = 10 μm.

**Figure 4:**
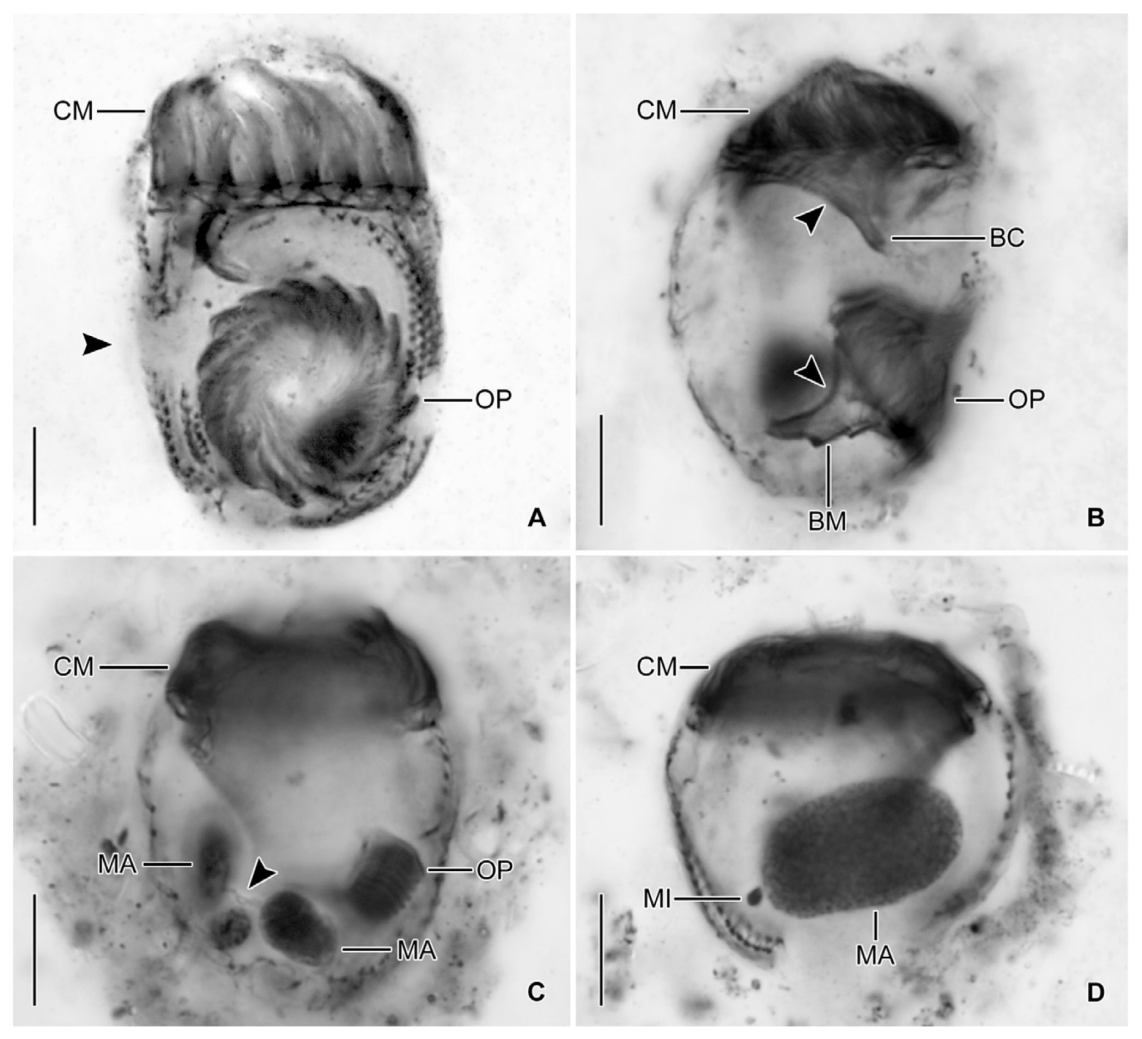
*Antetintinnidium mucicola* nov. gen., nov. comb., dividers from the North Atlantic after protargol staining. (**A**) Ventral view of a late divider showing the future division furrow (arrowhead; stacked images). (**B**) Optical longitudinal section of a late divider showing the endoral membranes (arrowheads) and buccal cavities of the proter and opisthe. (**C**) Middle divider showing the macronucleus nodules connected by a thin isthmus (arrowhead). (**D**) Postdivider with one huge macronucleus nodule and adjacent micronucleus. BC = buccal cavity; BM = buccal membranelle; CM = collar membranelles; MA = macronucleus nodule/s; MI = micronucleus; OP = oral primordium. Scale bars = 10 μm.

Adoral zone of membranelles closed, 20–24 μm in diameter, perpendicular to the main cell axis in contracted cells. Invariably 16 collar membranelles, whose polykinetids (bases) extend almost horizontally on the top of the peristomial rim, form a closed circle (Fig. 1A–F, 3C, 4A). One collar membranelle elongated into the buccal cavity containing one buccal membranelle as recognised in a few properly orientated cells or oral primordia of late dividers (Fig. 4B). Collar membranelles about 35 μm long, extend obliquely anteriorly in swimming cells, while motionless and bent towards the centre of the peristomial field, forming a cone in contracted cells (Fig. 2A, C). A conspicuous network of argyrophilic fibres links the polykinetids of the collar membranelles (Fig. 3A, B): (i) argyrophilic fibres connect the distal and proximal ends of the membranelles; (ii) a circular, horizontally orientated fibre extends in the centre of the peristomial rim; and (iii) two fibres each commence at the distal ends of the membranelles and extend obliquely rightwards and leftwards, terminating in the circular fibre. Pharyngeal fibres originate in the buccal vertex and extend obliquely posteriorly. Course of endoral membrane unique and difficult to assess (Fig. S2): the membrane commences in a deep dorsal invagination of the peristomial field, performs a rough semi-circle (in top-view) in a furrow between the peristomial rim and the vaulted peristomial field, terminating in the buccal cavity. Conspicuously long argyrophilic structures (possibly cilia) originate in the endoral membrane and extend parallel to the vaulted peristomial field.

#### Ontogenesis

Since the cells are rather small and only few dividers were available in the protargol slides, ontogenesis could not completely be reconstructed. The oral primordium forms apokinetally in a subsurface pouch. In early dividers, it is situated underneath the left half of the unciliated ventral stripe and the posterior portion of the last kinety (Fig. 1B, 3A). During its further development in middle and late dividers, it increases in volume and extends mostly rightwards (Fig. 1D), until it occupies the posterior two thirds of the unciliated ventral stripe between the first and the last kinety (Fig. 1E, 4A, B).

The two macronucleus nodules are connected by a thin isthmus in early to late dividers (Fig. 3D, 4C and Movies S1, S2). In these dividers, the replication bands traverse the nodules, while the oral primordium develops and increases in size. In very late dividers, the two nodules fuse to one irregular mass (Movie S3) situated underneath the fully developed oral primordium (Movie S4). Subsequently, the macronuclear mass splits into two nodules which stay connected by a thin isthmus. Then, their position in the cell changes by a joint rotation of both nodules, whereby one nodule becomes almost vertically orientated underneath the oral primordium in the posterior cell portion (Fig. 4A) and the other nodule achieves an approximately horizontal orientation in the anterior cell portion. Early post-dividers have one macronucleus nodule, which is larger than those of late dividers. The distinct homogenous granulation of this big nodule (Fig. 4D) suggests the imminent division, reconstructing the interphasic nuclear apparatus. The micronucleus division could not be observed.

**Figure 5:**
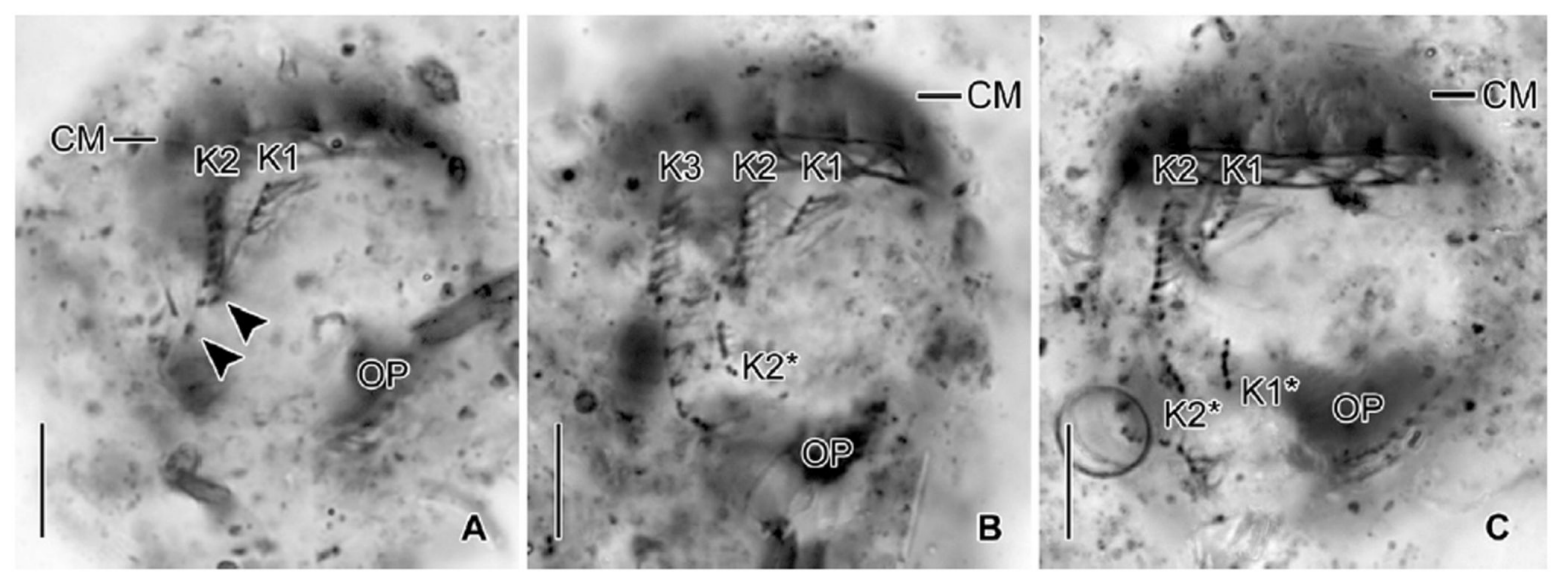
*Antetintinnidium mucicola* nov. gen., nov. comb., ventral views of early dividers from the North Atlantic after protargol staining. (**A**) Single dikinetids (arrowheads) are somewhat separated from the posterior ends of kineties 1 and 2. (**B, C**) The distance between the proter’s and opisthe’s fragments of kineties 1 and 2 increased, while additional kinetids proliferated primarily in the opisthe’s fragments. CM = collar membranelles; K1–3 = kineties 1–3 of the proter; K1* and K2* = kineties 1 and 2 of the opisthe; OP = oral primordium. Scale bars = 10 μm.

New somatic basal bodies are generated by intrakinetal proliferation. In the long kineties, the separation of the posteriormost dikinetids forming the origin of the opisthe’s fragments is not recognisable in early and middle dividers owing to their distinct curvature in the posterior cell portion; only in already elongated late dividers, the opisthe’s fragments are distinctly separated by a broad and unciliated horizontal stripe, the position of the future division furrow (Fig. 1E, 4A, B). In the posteriorly shortened first and second kineties, the successive separation of a single dikinetid from the posterior end of the proter’s rows is recognisable in early dividers (Fig. 5A). Since the single dikinetid from the first kinety is on the cell surface directly above the buccal cavity, it is difficult to observe. In late early dividers, already short fragments for the opisthe are found at the level of the oral primordium and thus distinctly apart from the proter’s fragments (Fig. 5B, C). Lorica formation or splitting as described by Reck (1988) were neither observed in live specimens nor recognisable in the stained material.

#### Observations on North Sea specimens

**Figure 6:**
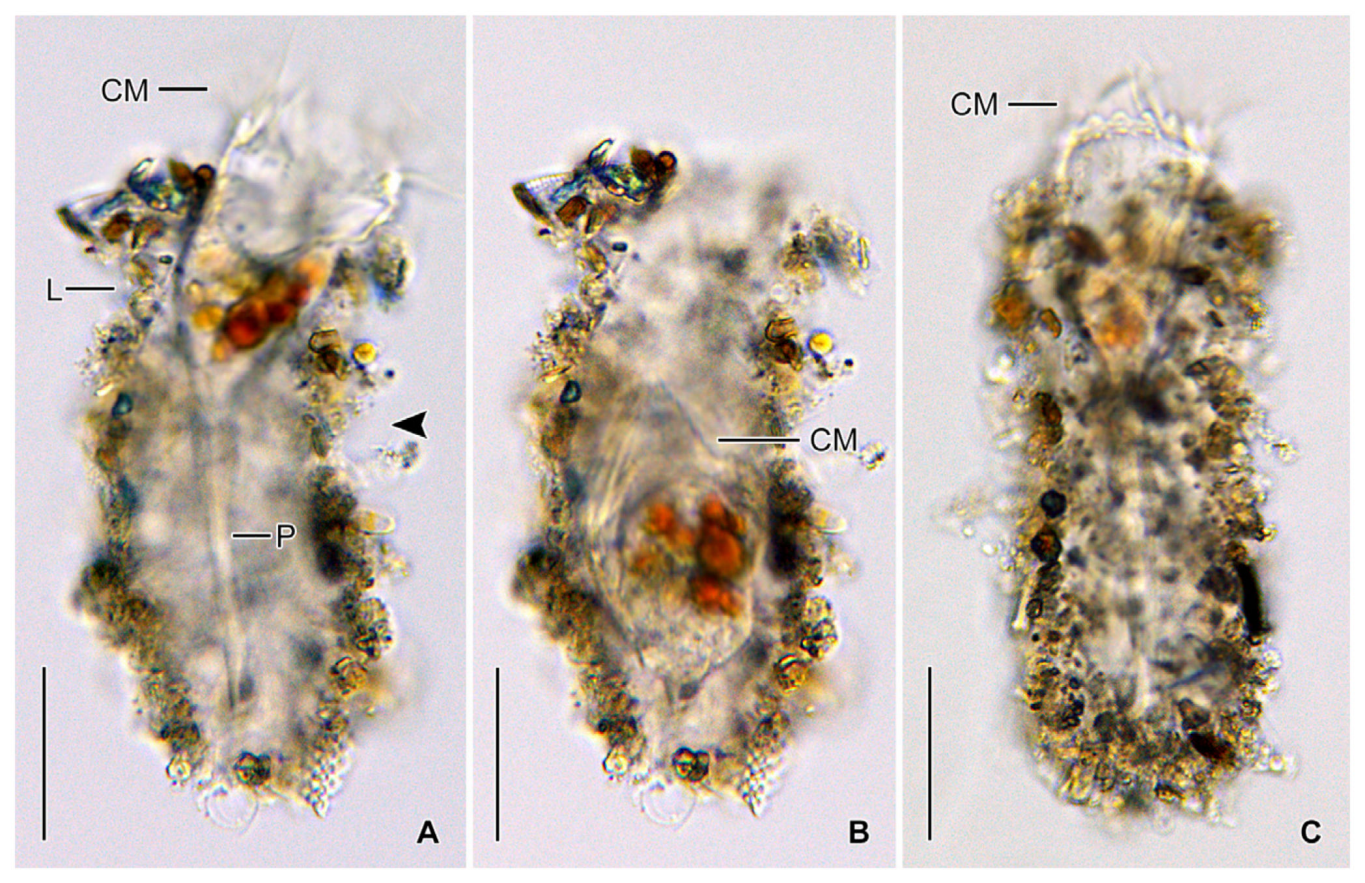
*Antetintinnidium mucicola* nov. gen., nov. comb., live specimens from the North Sea. (**A, B**) Same specimen in extended and retracted state. Note the shallow lateral concavity of the lorica (arrowhead). (**C**) Extended specimen with distinct intermembranellar ridges. CM = collar membranelles; L = lorica; P = peduncle. Scale bars = 30 μm.

The North Sea specimens (Fig. 6A–C) perfectly match those from the Chesapeake Bay in their morphology. The soft lorica is 95–115 μm in length and has an opening diameter of 28–34 μm. The cell proper measures about 35 × 31 μm in contracted live specimens and is about 50 μm long in the extended state. The peduncle is up to 70 μm long and attached to the bottom of the lorica. In the posterior cell portion, a couple of reddish/brownish inclusions (about 9 μm across), most likely food vacuoles, are visible in the otherwise colourless cytoplasm. Neither a contractile vacuole nor a cytopyge are recognisable. Several longitudinal and distantly arranged kineties with evenly spaced cilia extend on the dorsal side. The collar membranelles are up to 37 μm long and separated by distinct ridges (Fig. 6C).

#### Cladistic analyses

The cladistic analyses yielded 69 parsimony informative characters and 54,510 most parsimonious trees (L = 186, Ci = 0.64, Ri = 0.9). The consistency (Ci) and retention (Ri) indices are a measure for the phylogenetic information content ranging from 0 to 1, where 0 equals a lot of homoplasies and 1 equals perfect congruence among characters/between characters and the tree (Yang 2014). The tree length (L) is the sum of all character state changes, and the most parsimonious tree has the least length required to explain the mapping of all character state changes. The strict consensus tree (Fig. S1, left tree) obtained after calculation of the Bremer support values is longer (L = 200) but has similar consistency (Ci = 0.6) and retention indices (Ri = 0.88). Similar values are also attained for the 50% majority-rule consensus tree (Fig. S1, right tree; L = 202, Ci = 0.59, Ri = 0.88). Although both consensus trees show a slightly different topology regarding *A. mucicola* nov. gen., nov. comb. (polytomy vs. bifurcation), the species is invariably separated from *Membranicola*, *Tintinnopsis cylindrata*, and the *Tintinnidium* species.

#### Analyses of GenBank data

The congruent lorica morphologies and sizes in our specimens and those sequenced by Santoferrara et al. (2013) from the Northwest Atlantic and Zhang et al. (2017) from the Yellow Sea indicate conspecificity (see section ‘Comparison with further populations’), although no own gene sequences are contributed in the present study for comparison.

**Figure 7:**
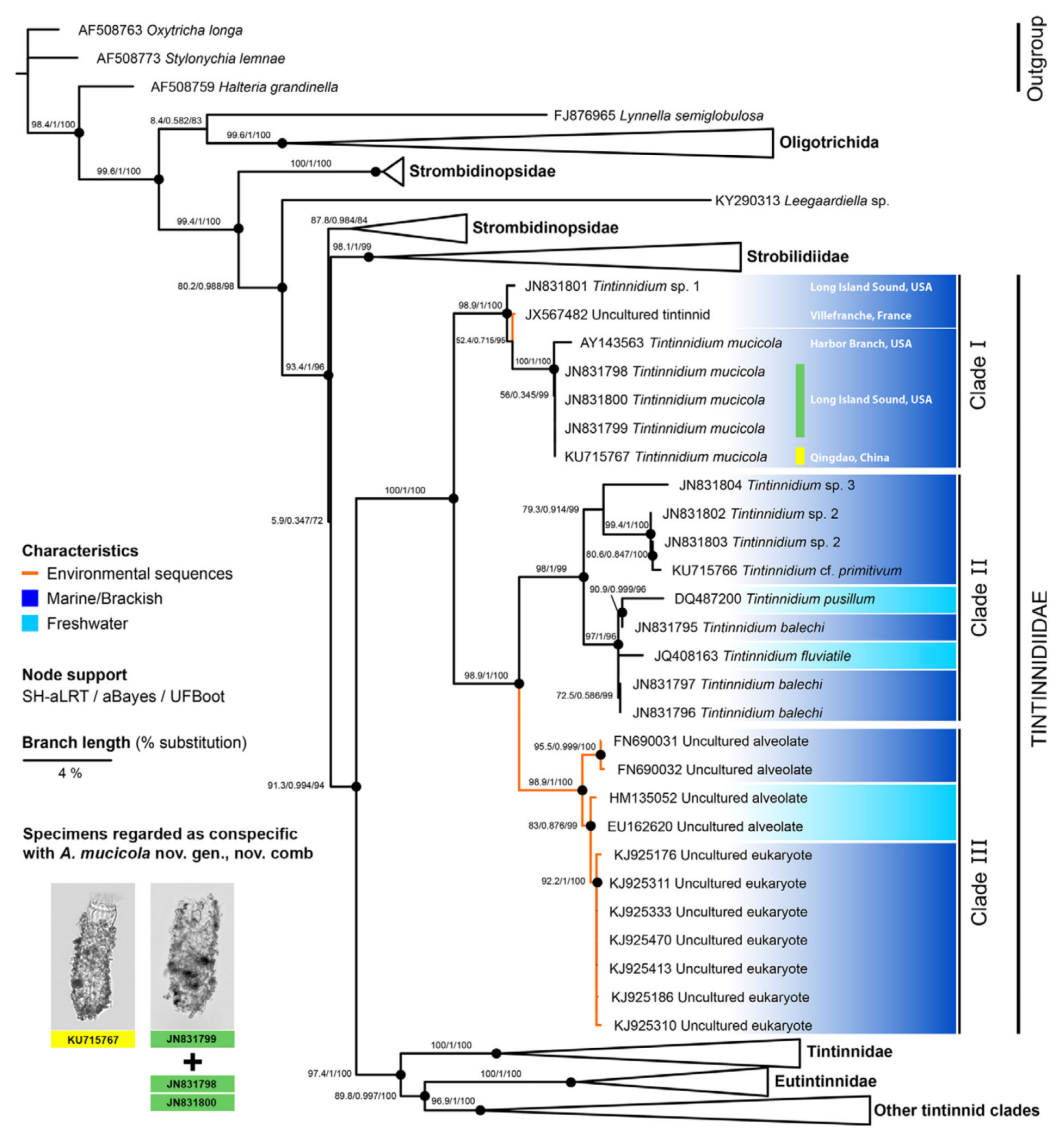
Phylogenetic relationships of taxa within the family Tintinnidiidae (part of the maximum likelihood tree of oligotrichid, choreotrichid, environmental, and outgroup 18S rDNA sequences retrieved from GenBank; Table S2). Nodes are only regarded as statistically supported (black circles) when the SH-aLRT values are ≥ 80% and the UFBoot values are ≥ 95% (Minh et al. 2013). Colours code the origin of the sequences, i.e. from marine/brackish (dark blue) and freshwater (light blue) habitats. Unidentified environmental sequences are marked by orange branches. Accession numbers of sequenced specimens regarded as conspecific with *Antetintinnidium mucicola* nov. gen., nov. comb. are highlighted with a yellow bar (micrograph from Zhang et al. 2017) and a green bar (Santoferrara et al. 2013; micrograph of a sequenced specimen kindly provided by L. F. Santoferrara).

The consideration of all available sequences from identified or unidentified specimens in the analyses provides a detailed insight into the genetic diversity of the Tintinnidiidae and their phylogenetic placement. The maximum likelihood tree of the 18S rDNA sequences (Fig. 7) fully supports the family Tintinnidiidae as monophyletic sister group to the remaining tintinnids. The family can be divided into three statistically supported main clades. Clade (I) comprises all available *T. mucicola* sequences. Those from the Northwest Atlantic (JN831798–JN831800) are identical to the one from the Yellow Sea (KU715767), and the first sequence for this species deposited in GenBank from the Indian River in Florida, USA (AY143563), is very similar to them (p-distance 0.2%). Additionally, one environmental sequence and one sequence of an unidentified tintinnidiid species fall into clade I (p-distances 1.5% and 1.8%, respectively). The two other clades form a well-supported sister group to clade I. Clade II comprises sequences from unspecified marine/brackish tintinnidiid species (JN831802–JN831804, KU715766), from the freshwater species *T. fluviatile* and *T. pusillum*, and the marine/brackish species *T. balechi*. Clade III exclusively comprises environmental sequences obtained from fresh- and marine/brackish waters. The pairwise distances of *T. mucicola* sequences to those of clades II and III range from 4.5% to 4.9% and about 4.1%, respectively. Comparisons of *T. mucicola* sequences with sequences of the Tintinnidae and Eutintinnidae revealed distances of 5.8–8.9% and 8.0–9.3%, respectively.

## Discussion

### Justification of populations’ conspecificity

Specimens with congruent lorica morphologies and sizes were collected within a comparatively short distance in the Chesapeake Bay (in vivo data: Annapolis Harbor; protargol-stained specimens: 130 km apart from Annapolis towards the estuary mouth) in different years and were recorded previously in this region (Dolan 1991). These records from different years suggest a common occurrence of the species in the Chesapeake Bay. Confusion with other *Tintinnidium* species is less likely owing to the distinctness of *A. mucicola* nov. gen., nov. comb. in lorica morphology and size (see section ‘Comparison with *Tintinnidium* species’).

### Comparison with original description

**Figure 8:**
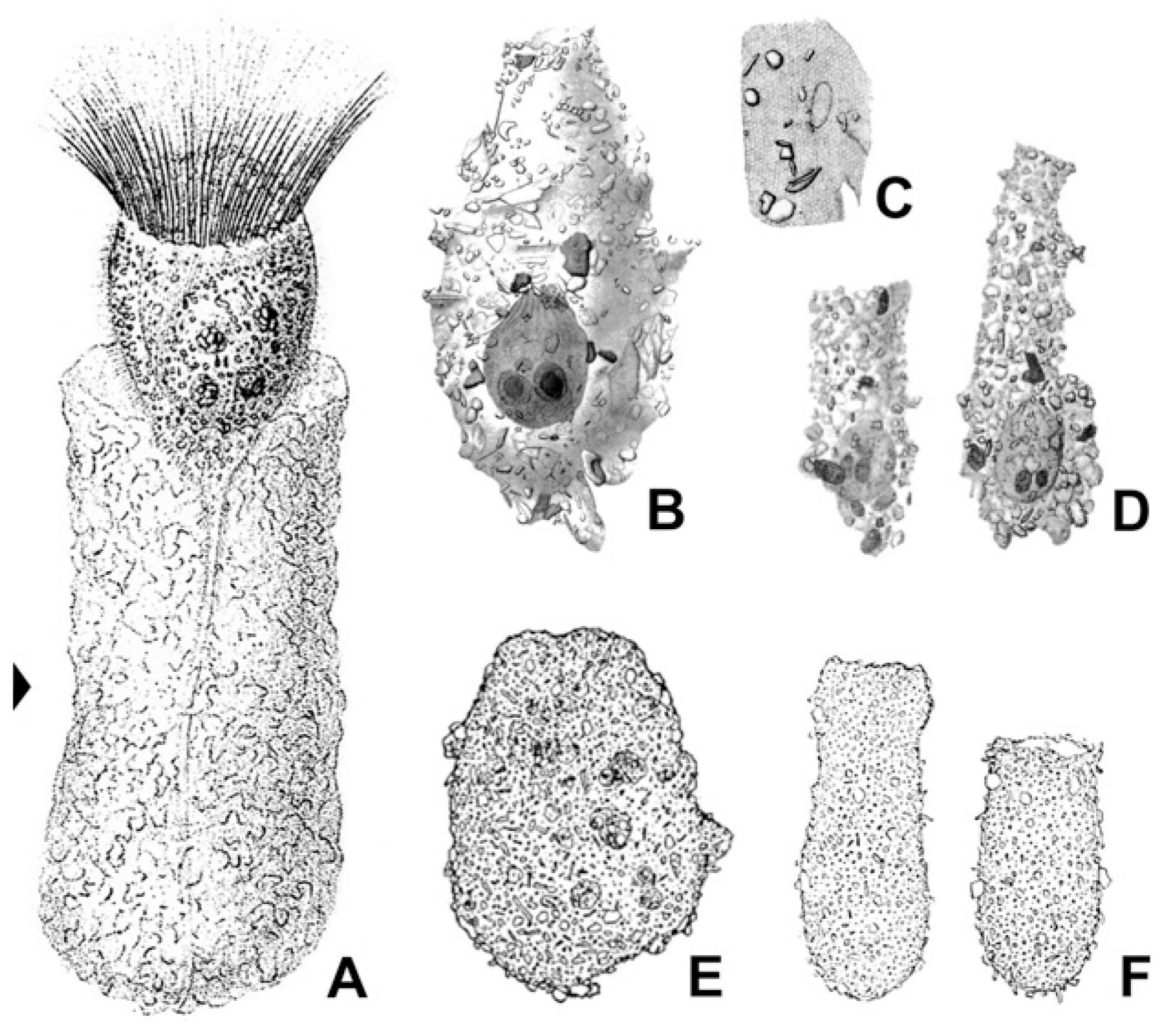
Illustrations of *Tintinnidium mucicola* from the literature matching our specimens in lorica shape (A, F) or having deviating lorica shapes and/or matrix structures (B–E). (**A**) Original illustration of a North Sea specimen (Claparède and Lachmann 1858). Size not mentioned, but only magnification ion of objective used. Note the shallow lateral concavity of the lorica (arrowhead). (**B–D**) Large (130–240 μm long) flask-shaped lorica (B) with fine alveolate matrix structure (C) and short, possibly damaged loricae (D) from the Baltic Sea (Brandt 1906, 1907). (**E**) Broadly ellipsoidal lorica 100–190 × 50–160 μm in size with opening diameter of 30–50 μm from the Palao Islands, equatorial West Pacific (Hada 1938). (**F**) Loricae 75–100 × 30–33 μm in size from the Akkeshi Bay, North-west Pacific (Hada 1937).

*Tintinnidium mucicola* was described by Claparède and Lachmann (1858) as a member of the genus *Tintinnus* Schrank, 1803. Its original description from the North Sea is based on live observations only (Fig. 8A) and matches the specimens from the neotype population in the following features: (i) the gelatinous structure of the lorica as well as its asymmetry; (ii) the proportions of the lorica, cell proper, peduncle, and membranelles; and (iii) the indication of intermembranellar ridges. Instead of providing measurements in the original description, Claparède and Lachmann (1858) mentioned a general magnification factor, which is hardly applicable for inferring precise dimensions from their figure. Hence, the lorica length of 170 μm inferred by Saville-Kent (1881), using exclusively the original description, is questionable. Nevertheless, the original description and illustration contain sufficient information, justifying the assumption of conspecificity with the North Atlantic specimens described here.

### Comparison with further populations

Under the name *Tintinnidium mucicola*, specimens with soft, posteriorly closed loricae are subsumed in the literature, although revealing a considerable variability in shapes and sizes. Specimens matching ours in lorica size and shape were found in the North Sea (Tempelman and Agatha 1997), the Northwest Atlantic (Brownlee 1977; Dolan 1991; Santoferrara et al. 2013), and the West Pacific (Hada 1937), including the Yellow Sea (Zhang et al. 2017).

The specimens depicted by Small and Lynn (1985) and Laval-Peuto (1994) are more or less modified line drawings from Brownlee’s (1977) unpublished Master Thesis and are not accompanied by descriptions and measurements. The somatic ciliary pattern matches that of our specimens perfectly, except for the course of the last kinety, which is entirely longitudinal and not curved in its anterior portion, extending parallel to the zone of adoral membranelles. The unpublished morphometric data of specimens from Delaware (USA; 38°51′N, 74°48′W; Brownlee 1977), however, fit very well, suggesting that the curvature might have been overlooked.

The lorica representative for the sequenced specimens (the genetic material was extracted from about 50 cells) from the Indian River in Florida, USA, seems to be strongly deformed and thus does not allow a morphological comparison (Strüder-Kypke and Lynn 2003). The single specimen from the Yellow Sea sequenced by Zhang et al. (2017) is congruent with the specimens in the present redescription: (i) the lorica widths/opening diameters are 35 μm (single specimen, Yellow Sea) and 33–50 μm (*A. mucicola* nov. gen., nov. comb., North Atlantic) and (ii) the loricae are slightly asymmetric due to a shallow lateral concavity observed in live specimens (cp. Fig. 7 with Fig. 1A, 2A). Likewise, the three sequenced specimens collected from the Long Island Sound, Northwest Atlantic, by Santoferrara et al. (2013; Fig. 7) are highly similar in their lorica dimensions (59–117 × 35–49 μm) to *A. mucicola* nov. gen., nov. comb. (69–107 × 33–50 μm). Therefore, we suggest linking the sequences of the specimens identified as *T. mucicola* by Zhang et al. (2017; KU715767) and by Santoferrara et al. (2013; JN831798–JN831800) with the redescription given in this study via the congruent lorica morphologies and sizes indicating conspecificity. Please, note that this assumption is based on a suboptimal combination of materials taken at different sites and at different times.

A few descriptions of specimens from the North Sea (Lauterborn 1894; Merkle 1909) and the Baltic Sea (Brandt 1906, 1907; Merkle 1909) depict loricae which are not cylindroidal as in the original description but posteriorly broadened, resembling a flask-shaped pouch (Fig. 8B). Specimens with this type of lorica apparently co-occurred with the typical form in different quantities. Due to a similar agglutination of particles and the gelatinous nature of their loricae, they were also identified as *T. mucicola*, although their loricae are quite large, ranging from 130–240 μm in length and 50–63 μm in width (Brandt 1906, 1907; Lauterborn 1894). Furthermore, the fine alveolate structure (Fig. 8C) of their lorica matrix is not present in the loricae of *A. mucicola* nov. gen., nov. comb. from the North Atlantic and North Sea (this study). Besides the fl k-shaped loricae, Brandt (1906, 1907) found irregular or deformed cylindroidal morphotypes (Fig. 8D).

Hada’s (1938) *T. mucicola* from lagoons of the West Caroline Islands, Palao (tropical West Pacific), differ in lorica shape and size from our specimens (Fig. 8E). He describes a broadly ellipsoidal morphotype measuring 100–190 μm in length, 50–160 μm in width, and 30–50 μm in the opening diameter. This morphotype distinctly differs from the specimens the author previously collected in the Akkeshi Bay, Japan (lorica length 75–100 μm, opening diameter 30–33 μm; Fig. 8F; Hada 1937). The latter loricae are similar to our specimens.

The diversity in lorica morphologies and sizes displayed by the studies from the North Sea (Lauterborn 1894; Merkle 1909) and the Baltic Sea (Brandt 1906, 1907; Merkle 1909) indicates that the specimens investigated are probably not conspecific. This is particularly supported by the differences in the opening diameters, a character which is known to constitute a less variable and thus generally more reliable taxonomic feature for delimiting congeneric tintinnid species (Laval-Peuto and Brownlee 1986).

The issue of regarding specimens with deviating loricae as conspecific and the resulting broadening of the species circumscription became serious when a flask-shaped lorica (Fig. 8B), a redraw of a figure from Brandt (1906), was included as representative of *T. mucicola* in the conspectus of Kofoid and Campbell (1929). The improved species diagnosis given here is therefore restricted to the type and neotype populations only (see section ‘Taxonomic Summary’).

### Comparison with *Tintinnidium* species

Saville-Kent (1881) established the genus *Tintinnidium* for sedentary tintinnids with mucilaginous loricae. This diagnosis was emended by Entz (1884) and von Daday (1887) by restricting it to the gelatinous composition of the lorica as the main distinguishing feature because *T. fluviatile,* the ‘typical representative’ of the genus, is mostly found as planktonic form. Accordingly, von Daday (1887) transferred *Tintinnus mucicola* to the genus *Tintinnidium.* The valid type species of the genus is *Tintinnus fluviatilis* Stein, 1863, and *Tintinnidium* Saville-Kent, 1881 is the type of the family Tintinnidiidae Kofoid and Campbell, 1929 (ICZN 1970; Tappan and Loeblich 1967). The genus *Tintinnidium* Saville-Kent, 1881 is now mainly characterised by its soft and aborally closed cylindroidal lorica which is covered by a wide variety of foreign particles to differing degrees.

Since its establishment more than 130 years ago, it always comprised freshwater and marine/brackish species. Currently, five marine/brackish [*T. balechi* Barría de Cao, 1981; *T. incertum* Brandt, 1906; *T. mucicola* (Claparède and Lachmann, 1858) von Daday, 1887; *T. neapolitanum* von Daday, 1887; and *T. primitivum* Busch, 1923 a supposed synonym of *T. incertum* (Hofker 1931; Kofoid and Campbell 1929)] and three freshwater species are known [*T. fluviatile* (Stein, 1863) Saville-Kent, 1881; *T. pusillum* Entz, 1909; and *T. semiciliatum* (Sterki, 1879) Saville-Kent, 1881]; *Tintinnopsis cylindrata* Kofoid and Campbell, 1929 should also be assigned to the genus based on its somatic ciliary pattern but its transfer has to await clarification of the taxonomic uncertainties concerning the type species *Tintinnopsis beroidea* (Foissner and Wilbert 1979; Laval-Peuto and Brownlee 1986; Petz and Foissner 1993). The marine/brackish species mentioned above differ from *A. mucicola* nov. gen., nov. comb. in lorica shape and size: (i) *T. balechi* has a distinctly narrower lorica (15–26 μm wide), (ii) *T. incertum* has a comparatively long lorica (240–260 μm) and shows an alveolar wall texture, and (iii) *T. neapolitanum* is characterised by a pyriform lorica (117 × 45 μm in size) with a distinct collar.

**Figure 9:**
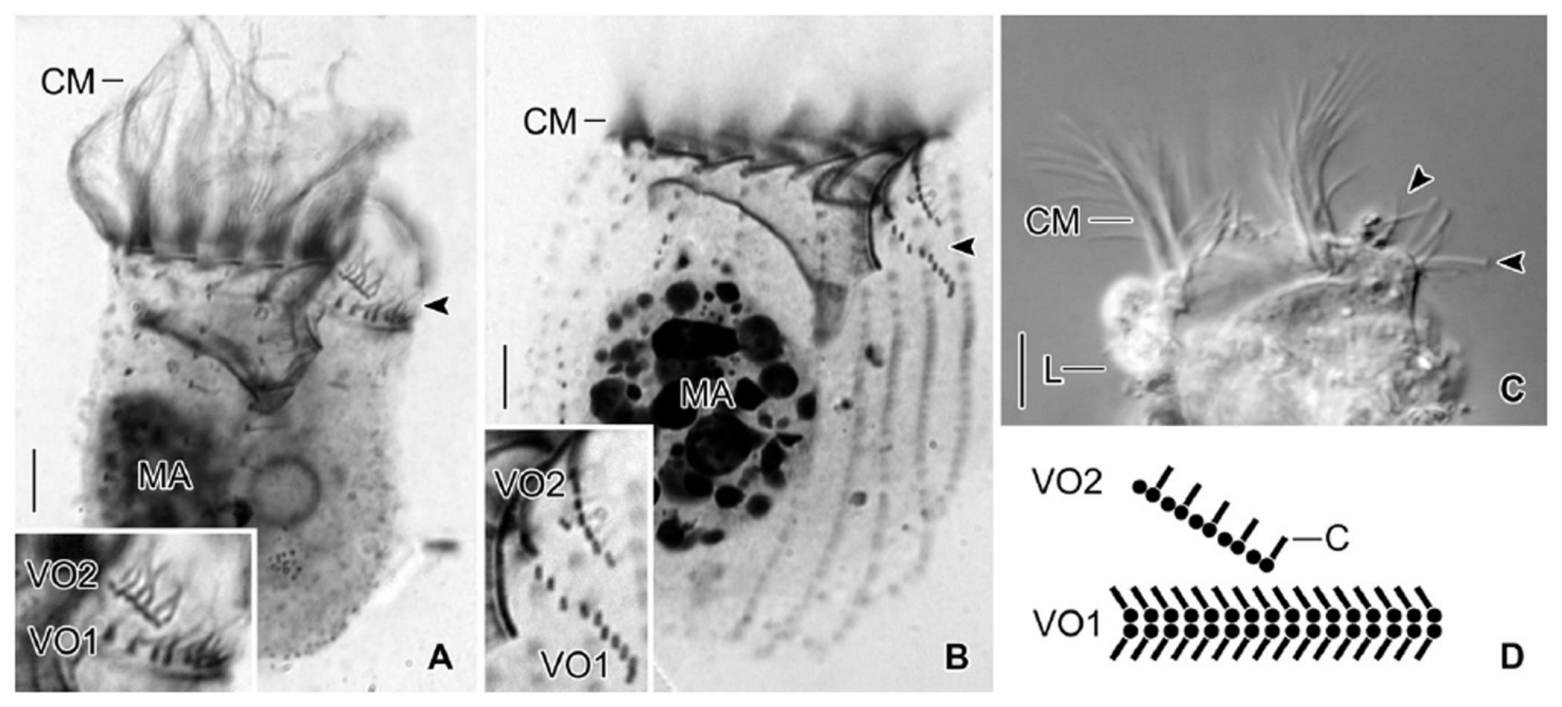
Ventral organelles in freshwater *Tintinnidium* species. (**A, B**) Right lateral views of *T. pusillum* (A) and *T. semiciliatum* (B) and enlarged details depicting the ventral organelles (arrowheads) after protargol staining. (**C**) Living freshwater *Tintinnidium* specimen showing the long and stiff cilia of the ventral organelles (arrowheads). (**D**) Scheme of ventral organelles. C = cilia; CM = collar membranelles; L = lorica; MA = macronucleus nodule; VO1 = ventral organelle 1; VO2 = ventral organelle 2. Scale bars = 10 μm.

The first detailed investigation of the cell morphology and, especially, of the somatic ciliature in freshwater *Tintinnidium* species was conducted by Foissner and Wilbert (1979). They already noted that identification of their specimens was difficult based on the information given in the original and subsequent descriptions because of some considerable differences. These mainly concerned the lorica sizes, but also the preliminary observations of the cell features. Nevertheless, the authors regarded the opening diameter of the loricae as the main distinguishing feature among congeners. The freshwater species redescribed by Foissner and Wilbert (1979) were identified as *T. fluviatile*, the type species of the genus (ICZN 1970; Kofoid and Campbell 1939; Tappan and Loeblich 1967), and *T. pusillum*. Although the two species differ in the sizes of their loricae, they share some cell features: (i) one ellipsoidal macronucleus and one micronucleus; (ii) somatic kineties consisting exclusively of dikinetids; (iii) a distinct unciliated ventral stripe; and (iv) two specialised ciliary structures, the ventral organelles, located on a ventral bulge directly posteriorly to the membranellar zone and composed of densely spaced dikinetids (Fig. 9A–D).

Ventral organelle 1 is more or less perpendicularly orientated to the main cell axis and thus parallel to the membranellar zone. It consists of 13–16 dikinetids with long and stiff cilia originating from each dikinetidal basal body (Fig. 9C). Interestingly, the dikinetids are perpendicular to the kinety axis, while all other somatic kinetids are parallel to the kinety axes. An argyrophilic fibre extends horizontally underneath the organelle. Ventral organelle 2 is anterior to ventral organelle 1. It is shorter than organelle 1, anti-clockwise inclined, and comprises usually five, in interphasic stages up to six dikinetids arranged parallel to the kinety axis (Petz and Foissner 1993). Only the posterior basal body of each dikinetid has associated a long and stiff cilium. The orientation described here is based on re-investigations of the type slides, while the figures depicted in Foissner and Wilbert (1979) are mirror-inverted. The most conspicuous difference between the ventral organelles and the remaining somatic ciliature in these species and tintinnid kineties in general is their de novo origin (Petz and Foissner 1993). The above mentioned characteristics and those observed in *T. semiciliatum* were added to the genus diagnosis of *Tintinnidium* by Agatha and Strüder-Kypke (2007) to include both, information on the lorica and cell morphology.

The redescription of *A. mucicola* nov. gen., nov. comb. is the first treating a marine/brackish member of the Tintinnidiidae. The species distinctly differs from the fresh-water species in some genus–characteristic morphological features. At first glance, the first and second kineties of *A. mucicola* nov. gen., nov. comb. represent promising structures for hypothesising homology with the ventral organelles. However, these kineties proliferate basal bodies intrakinetally as all other kineties and thus do not originate de novo. Additionally, the first kinety exhibits a different orientation (clockwise vs. anti-clockwise inclined), although it matches ventral organelle 2 in size and structure. Kinety 2 differs from ventral organelle 1 also in its course (longitudinal vs. more or less horizontal), the orientation of the dikinetids (parallel vs. perpendicular to the kinety axis), and the number of cilia per dikinetid (one vs. two). In the freshwater *Tintinnidium* species, there are also no kineties resembling the last kinety in its distinct curvature and the penultimate kinety in its distinct anterior shortening. The last kinety is also not homologous to the ventral kinety occurring in the ciliary patterns of the other tintinnid families, mainly because it is exclusively dikinetidal (vs. monokinetidal) and located on the left side of the oral primordium (vs. the right side). Beyond the obvious absence of ventral organelles, *A. mucicola* nov. gen., nov. comb. invariably has two macronucleus nodules (vs. one nodule in freshwater *Tintinnidium* species).

### Phylogeny and diversity of the Tintinnidiidae

The present phylogenetic analyses of morphological and molecular data clearly demonstrate distinct differences between *A. mucicola* nov. gen., nov. comb. and the *Tintinnidium* species including the type species *T. fluviatile*. These findings are supported by recent molecular studies displaying similar tree topologies regardless of the ribosomal sequences analysed (18S, 28S rDNA, concatenated datasets) and the tree building algorithms applied (Bachy et al. 2012; Santoferrara et al. 2013, 2015; Zhang et al. 2017). Likewise, *A. mucicola* nov. gen., nov. comb. differs from the monotypic genus *Membranicola* Foissner et al., 1999 by possessing a lorica with a broadly rounded posterior end (vs. posteriorly closed by a subterminal membrane) and the ventral organelles (absent vs. present).

The cell morphology of *A. mucicola* nov. gen., nov. comb. encompasses several supposedly plesiomorphic characters that might have already been present in the last common tintinnid ancestor (Agatha and Strüder-Kypke 2007). First of all, the somatic ciliature exhibits a uniform kinetid structure and no distinct separation into a right and left ciliary field. Instead, the kineties are widely and equidistantly spaced, except for the last three kineties. Agatha and Strüder-Kypke (2007) used the different spacing of kinetids and kineties on both cell sides shown in the kinetal maps of Foissner and Wilbert (1979) and Blatterer and Foissner (1990) for defining right and left ciliary fields separated by an unciliated ventral stripe. However, our re-investigation of the type slides of *T. pusillum* and *T. semiciliatum* revealed that the distances of kinetids and kineties become gradually smaller in clockwise direction. Thus, these species, like *A. mucicola* nov. gen., nov. comb., do not possess a right and left ciliary field. Accordingly, these fields probably occurred later in the tintinnid evolution, namely, only in species with a ventral kinety, in which the fields are also dorsally separated by an unciliated stripe and subsequently by dorsal kineties. Examples for these patterns can be found in the extant genera *Nolaclusilis* (unciliated dorsal stripe) and *Eutintinnus* (dorsal kineties), respectively.

Beyond the rather homogeneous spacing of kineties and kinetids in *A. mucicola* nov. gen., nov. comb., the other presumably ancestral characters include: (i) dikinetidal somatic kineties; (ii) two macronucleus nodules; (iii) an adoral zone of membranelles with an always perpendicular orientation; (iv) some elongated collar membranelles of the closed adoral zone extending into the buccal cavity; (v) a contractile peduncle; and (vi) an enantiotropic division mode with a hypoapokinetal stomatogenesis in a pouch and an intrakinetal proliferation of basal bodies. *Antetintinnidium mucicola* nov. gen., nov. comb. displays not only plesiomorphic features but also a derived character, namely, somatic dikinetids that have a cilium associated only with each posterior basal body. According to the hypothesis of kinetid transformation suggested by Agatha and Strüder-Kypke (2014), the plesiomorphic state in somatic kinetids of Oligotrichea is a dikinetid with a cilium only at the anterior basal body. Next, the posterior basal body became ciliated, too, and the anterior cilium was subsequently lost, generating the kinetid type found in *A. mucicola* nov. gen., nov. comb. The ventral organelles, however, constitute a synapomorphy of the genera *Tintinnidium* and *Membranicola*, especially when their de novo origin has been confirmed. According to the lack of these special organelles in *A. mucicola* nov. gen., nov. comb. and the considerable genetic divergence of this species, a new genus is established and the diagnosis of the family Tintinnidiidae is improved (see section ‘Taxonomic Summary’).

The genetic diversity within the family Tintinnidiidae indicates that it probably comprises more species or even genera than currently known. Particularly, clades II and III contain several sequences of unidentified specimens distinctly diverging from the sequences of the known species (Santoferrara et al. 2013; Zhang et al. 2017). While the softness of the lorica seems to be a reliable feature characterising the family, species identification is often hampered by the easily deformed loricae, the inconspicuousness of the lorica matrix material, and the distinct influence of the agglutinated particles on the lorica outline. This becomes more serious with decreasing lorica size. Unfortunately, descriptions of cell morphology are lacking for most species genetically analysed. One example is *T. balechi*, a marine/brackish species, which had been described from an Argentinian estuary based only on its lorica characteristics (Barría de Cao 1981). The grouping of its 18S rDNA sequences with those from the freshwater congeners *T. fluviatile* and *T. pusillum* in clade II suggests not only their close phylogenetic relationship (Fig. 7), but also the possession of the apomorphic ventral organelles.

The genus *Tintinnidium* comprises the subgenera *Tintinnidium* and *Semitintinnidium* (Agatha and Strüder-Kypke 2007). This subdivision is not recognisable in the molecular genealogies as the benthic *Tintinnidium (Semitintinnidium) semiciliatum* has not been sequenced as yet and the identification of *Tintinnidium (Tintinnidium) pusillum* cannot be verified.

The imbalance of morphological and molecular data is especially apparent when considering environmental sequences. So, clade III exclusively comprises sequences from limnetic and marine/brackish samples that can currently not be linked to any known species owing to the lack of morphological data and barcodes. Only by collecting further morphological and molecular data in integrative studies can the real diversity of the Tintinnidiidae be assessed.

### Occurrence and ecology

The biogeographical data on *A. mucicola* nov. gen., nov. comb. are rather scarce (Table S1). The conspecificity of the records substantiated by lorica illustrations and/or measurements is discussed above (see sections ‘Comparison with original description and further populations’). Substantiated records stretch over a period of 160 years and cover many different coastal regions (Fig. S3), namely, the Pacific Ocean (Hada 1937; Zhang et al. 2017), the Northwest Atlantic (Brownlee 1977; Dolan 1991; Santoferrara et al. 2013; Strüder-Kypke and Lynn 2003; this study), and the North Sea (Claparède and Lachmann 1858; Tempelman and Agatha 1997; this study). The majority of records are, however, uncorroborated, i.e., they do not provide enough evidence to prove the identification of the specimens. These records enlarge the distribution of *A. mucicola* nov. gen., nov. comb. to the coastal zones of the Baltic Sea, the Black Sea, the Mediterranean Sea, the Sea of Japan, the Indian Ocean, and the Western Arctic Sea.

According to the substantiated and most of the uncorroborated records, *A. mucicola* nov. gen., nov. comb. is restricted to neritic surface waters of the northern hemisphere. It is eurytherm, occurring at temperatures ranging from 3.5 °C (Hada 1937) to 21 °C (Zhang et al. 2017); our data are close to the upper limit (15–21 °C). The two uncorroborated records from the Indian Ocean (Anandakumar and Thajuddin 2013; Biswas et al. 2013) are exceptional, mentioning water temperatures of 26–32 °C; they mark the most southern report of the species. The most northern records are represented by the type locality, namely, the Fjord of Bergen in Norway (Claparède and Lachmann 1858), and a recent uncorroborated record from the Western Arctic Sea (Matsuno et al. 2014).

*Antetintinnidium mucicola* nov. gen., nov. comb. tolerates quite a broad spectrum of salinities ranging from oligohaline (2‰; Godhantaraman and Uye 2003) to euhaline (35‰; Zhang et al. 2017). The present data demonstrate an occurrence in mesohaline waters characterised by salinity changes typical of estuaries. Abundances of about 1,600 individuals per litre have been estimated for the Chesapeake Bay in spring (Dolan 1991). Further uncorroborated records report noticeable abundances of the species during spring and autumn in various geographical regions (Dolgopolskaia 1940; Graziano 1989; Monti et al. 2012; Yu et al. 2013).

## Taxonomic Summary

Class Oligotrichea Bütschli, 1887

Order Choreotrichida Small and Lynn, 1985

Suborder Tintinnina Kofoid and Campbell, 1929

Family Tintinnidiidae Kofoid and Campbell, 1929

**Remarks**. Previous diagnoses included the numbers of macronucleus nodules and collar membranelles as well as the rather simple somatic ciliary pattern (Laval-Peuto 1994) or are restricted to lorica features (Lynn 2008). Since the genus *Leprotintinnus* is excluded from the Tintinnidiidae based on molecular and preliminary morphological data (Zhang et al. 2017) and the present study provides a new somatic ciliary pattern, the family diagnosis necessitates an improvement. Please, note that *Tintinnopsis cylindrata* has a similar morphology but is not considered in the diagnosis (see above).

**Improved diagnosis**. Lorica usually cylindroidal, posteriorly closed by lorica wall or subterminal membrane; lorica wall soft, gelatinous, with agglutinated particles. One or two macronucleus nodules and one micronucleus. Somatic ciliature interrupted by distinct ventral stripe without cilia or merely ventral organelles, exclusively dikinetidal or with monokinetids in posterior third or half of kineties. Buccal membranelle indistinct or absent. In marine, brackish, and freshwater habitats; lifestyle mostly planktonic, rarely sessile.

**Included genera.** *Antetintinnidium* nov. gen., *Membranicola* Foissner, Berger, and Schaumburg, 1999, and *Tintinnidium* Saville-Kent, 1881.

### *Antetintinnidium* nov. gen

**Diagnosis**. Lorica cylindroidal, posteriorly closed by broadly rounded lorica wall. Two macronucleus nodules. Somatic kineties interrupted by unciliated ventral stripe; kineties exclusively composed of dikinetids each having associated a cilium only with the posterior basal body, all originate by intrakinetal proliferation of basal bodies. With buccal membranelle. Planktonic.

**ZooBank registration number**. 04DB5B70-54DF-4D5E-88C7-1ECC792DBBEF.

**Type species**. *Tintinnus mucicola* Claparède and Lachmann, 1858

**Etymology**. Composite of the Latin prefix *ante*- (“before in place or time”) and the genus name *Tintinnidium*, indicating a high similarity to that genus in lorica features, but displaying a more ancestral somatic ciliary pattern.

**Comparison with related genera**. The related genera *Tintinnidium* and *Membranicola* differ from *Antetintinnidium* nov. gen. by the two de novo originating ventral organelles. The genus *Membranicola* differs additionally by its tube-shaped lorica subterminally closed by a membrane (Foissner et al. 1999). The genus *Tintinnidium* is also distinguished by possessing a single macronucleus (vs. two nodules) and somatic kineties with some dikinetids having associated two cilia (vs. invariably with cilia only at the posterior dikinetidal basal bodies) or with monokinetids in the posterior third to half (vs. exclusively dikinetids).

### *Antetintinnidium mucicola* (Claparède and Lachmann, 1858) nov. gen., nov. comb

1858 *Tintinnus mucicola*—Claparède and Lachmann, Études sur les infusoires et les rhizopodes. *Mém. Inst. natn. génev.,* **5**: 209 + Vol. 5, Plate 18, fig. 12 (basionym).

1887 *Tintinnidium mucicola*—von Daday, Monographie der Familie der Tintinnodeen. *Mitt. zool. Stn Neapel,* **7**: 524 (new combination).

**Remarks**. Congruent lorica morphology unites the specimens collected at different times and at different sites in the Chesapeake Bay and Long Island Sound (Northwest Atlantic), from the North Sea, and from the Yellow Sea (Table S1; Fig. 1A, 2A, 6A, B, 7). Nevertheless, the following diagnosis is only based on the original description and specimens sampled in the Chesapeake Bay. A neotype is designated here owing to the severe inconsistencies regarding the species circumscription in the literature and the resulting taxonomic confusion. Particularly, the identification of *T. mucicola* based on morphotypes deviating from the original description and probably representing distinct species (Hofker 1931), necessitated a revision and neotypification after a detailed redescription.

The neotype specimen fits the original description. Physical type material very likely does not exist, as the original description is from the year 1858 and thus was published before methods generating permanent slides became available.

According to the rather wide distribution of the species, it seems justified to designate a neotype from a different site, especially, as both the type (Fjord of Bergen) and neotype localities (Chesapeake Bay) belong to the warm temperate region of the North Atlantic and are connected by oceanic currents (Table S1; Fig. S3).

Physical neotype material will be made available in a research collection (see below). The need for and problems with neotypification have already thoroughly been discussed by other authors (Corliss 2003; Foissner 2002; Foissner et al. 2002).

Note that no gene sequence of the species was obtained in the present study, but the following sequences are supposed to belong to conspecific specimens because of a congruent lorica morphology: KU715767 from the Yellow Sea (Zhang et al. 2017); JN831798, JN831799, and JN831800 from the Northwest Atlantic (Santoferrara et al. 2013).

**Improved diagnosis**. Lorica cylindroidal, about 85 × 42 μm in size. Cell in extended state elongate obconical, about 100 × 30 μm in size, in contracted state subspherical, about 35 × 30 μm in size in vivo, about 24 × 26 μm in size after protargol-staining. Usually 16 dikinetidal somatic kineties; first kinety short, clockwise inclined, with long motile cilia; second kinety posteriorly shortened, composed of densely spaced kinetids; last kinety usually longest row, bent leftwards in anterior portion, extending longitudinally in posterior portion; penultimate kinety shortened anteriorly. Invariably 16 collar membranelles, of which one extends into buccal cavity; one buccal membranelle. Marine and brackish waters.

**Type locality**. The species was first described by Claparède and Lachmann (1858) from the Fjord of Bergen at the Norwegian coast, North Sea. The neotype material is from the Chesapeake Bay (37°44′N, 76°11′W), an estuary at the east coast of the USA discharging into the North Atlantic.

**Neotype material**. The species is neotypified from the Chesapeake Bay, Maryland, USA. Slides with protargolstained material, including the neotype and further specimens are deposited with the relevant cells marked in the Biology Centre of the Museum of Upper Austria (LI) in 4040 Linz, Austria.

## Supplementary Material

Movie S4

Movie S3

Movie S2

Movie S1

Suppl file

## Figures and Tables

**Table 1:** Morphometric data on *Antetintinnidium mucicola* nov. gen., nov. comb. from the Chesapeake Bay (ML, USA)

Characteristics^a^	$\bar{x}$	M	SD	SE	CV	Min	Max	*n*
Lorica, total length^b^	83.9	84.0	11.4	3.2	13.5	69	107	13
Lorica, width/opening diameter	42.4	41.0	5.4	1.5	12.9	33	50	14
Cell proper, length	23.7	23.0	2.8	0.6	12.0	20	31	23
Cell proper, width	26.1	25.0	2.1	0.4	8.0	23	30	23
Cell proper, length:width ratio	0.9	0.9	0.1	0.0	7.4	0.8	1.1	23
Anterior cell end to buccal vertex, distance	9.4	9.5	0.6	0.2	6.9	8	10	14
Macronucleus nodules, length	6.3	6.0	1.0	0.2	15.2	5	8	26
Macronucleus nodules, width	5.1	5.0	–	–	–	5	6	26
Macronucleus nodules, number	2.0	2.0	0.0	0.0	0.0	2	2	26
Anterior cell end to macronucleus nodules, distance	10.1	10.0	1.9	0.4	18.6	6	14	22
Micronucleus, length	1.0	1.0	0.0	0.0	0.0	1	1	8
Micronucleus, width	1.0	1.0	0.0	0.0	0.0	1	1	8
Micronucleus, number	1.0	1.0	0.0	0.0	0.0	1	1	8
Somatic kineties, number	16.1	16.0	–	–	–	16	17	21
Kinety 1, length	4.0	4.0	0.0	0.0	0.0	4	4	18
Collar membranelles to kinety 1, distance	3.1	3.0	–	–	–	3	4	16
Kinety 1, number of dikinetids	4.3	4.0	–	–	–	4	5	16
Kinety 2, length	7.3	8.0	1.1	0.3	15.2	6	9	13
Collar membranelles to kinety 2, distance	2.3	3.0	1.0	0.3	42.2	1	3	12
Kinety 2, number of dikinetids	8.0	8.0	0.5	0.1	5.9	7	9	10
Kinety 3, length^c^	14.6	14.0	2.6	0.9	17.6	10	18	9
Collar membranelles to kinety 3, distance	1.0	1.0	0.0	0.0	0.0	1	1	9
Kinety 3, number of dikinetids per 10 μm	9.4	9.0	0.9	0.3	9.3	8	11	9
Kineties 4–14, length^c^	15.8	15.5	1.2	0.5	7.4	15	18	6
Collar membranelles to kineties 4–14, distance	1.0	1.0	0.0	0.0	0.0	1	1	11
Kineties 4–14, number of dikinetids per 10 μm	7.9	8.0	0.8	0.3	10.6	7	9	8
Kinety *n*−1, length^c^	13.1	13.0	1.9	0.7	14.2	11	16	7
Collar membranelles to kinety *n*−1, distance	8.2	8.0	1.0	0.3	12.4	6	9	15
Kinety *n*−1, number of dikinetids	12.0	12.0	0.6	0.3	5.3	11	13	6
Kinety *n*, length^c^	19.9	19.5	2.4	0.8	12.2	16	24	10
Collar membranelles to kinety *n*, distance	1.3	1.0	0.7	0.2	54.7	1	3	16
Kinety *n*, number of dikinetids per 10 μm	9.2	9.0	0.9	0.3	10.2	8	11	12
Adoral zone of membranelles, diameter	22.3	23.0	1.4	0.3	6.3	20	24	27
Collar membranelles, number	16.0	16.0	0.0	0.0	0.0	16	16	7
Elongated collar membranelles, number^d^	1.0	1.0	0.0	0.0	0.0	1	1	2
Buccal membranelles, number^d^	1.0	1.0	0.0	0.0	0.0	1	1	2

CV = coefficient of variation in %; M = median; Max = maximum; Min = minimum; *n* = number of individuals investigated; SD = standard deviation; SE = standard error of arithmetic mean; $\bar{x}$ = arithmetic mean.

a Data are based on protargol-stained, mounted and randomly selected specimens from field material. Measurements in μm.

b Reliable measurements difficult as the soft loricae usually have a deformed or damaged anterior portion.

c Length of kineties extending to the posterior polar area difficult to measure because of their distinct curvatures in the posterior cell portion.

d Elongated collar membranelle and buccal membranelle rarely visible due to horizontal orientation of peristomial rim and overlaying structures. The data are from oral primordia of two late dividers.
