# Supplementary material for: Redescription of Antetintinnidium mucicola (Claparède and Lachmann, 1858) nov. gen., nov. comb. (Alveolata, Ciliophora, Tintinnina)
Source: J Eukaryot Microbiol. Author manuscript; Available in PMC 2020 Sep 1. (PMC6736674; doi:10.1111/jeu.12728)
Supplement: Supplementary file 1 [file EMS83096-supplement-Suppl_file.pdf]

SUPPORTING INFORMATION

Redescription of *Antetintinnidium mucicola* (Claparède and Lachmann, 1858) nov. gen., nov. comb. (Alveolata, Ciliophora, Tintinnina) by Maximilian H. Ganser and Sabine Agatha

Fig. S1. Cladistic trees. Left tree: strict consensus with bootstrap/Bremer support values (length = 200, consistency index = 0.6, retention index = 0.88). Right tree: majority consensus with bootstrap values (length = 202, consistency index = 0.59, retention index = 0.88). The analyses included 68 taxa and 95 characters, of which 69 are parsimony informative. Only bootstrap values > 50% are shown. The introduction of the ventral organelles (black square) and the ventral kinety (black circle) are indicated on the respective branches. (*) *Tintinnopsis cylindrata* can only be assigned to the genus *Tintinnidium* when the taxonomic uncertainties concerning the type species *Tintinnopsis beroidea* are solved.

Fig. S2. Protargol-stained specimens of *Antetintinnidium mucicola* nov. gen., nov. comb. depicting the conspicuous endoral membrane. **A**. Specimen showing the long and densely arranged argyrophilic structures (arrowhead), possibly the cilia of the endoral membrane. **B-E**. Images of consecutive focal planes of the same specimen displaying the length and curvature of the endoral membrane and its associated distinctly argyrophilic structures. CM – collar membranelles, MA – macronucleus nodules. Scale bars = 10 µm.

Fig. S3. Biogeography of *Antetintinnidium mucicola* nov. gen., nov. comb. based on literature and own records. World map showing the type and neotype localities (purple stars), records supported by illustrations and/or measurements (green circles), uncorroborated records (yellow triangles), and records of all tintinnid species [light blue circles; data from Dolan and Pierce (2013)].

Table S1. Records of *Antetintinnidium mucicola* nov. gen., nov. comb. Categories (CAT): (1) From original type and neotype localities, (2) substantiated records, and (3) uncorroborated records

Table S2. List of 18S rDNA sequences used for calculation of maximum likelihood tree

Movie S1. Consecutive focal planes showing the protargol-stained neotype specimen from the ventral to the dorsal side.

Movie S2. Consecutive focal planes showing a protargol-stained paratype specimen from top to posterior polar view.

Movie S3. Consecutive focal planes showing the ventral side of a protargol-stained late divider.

Movie S4. Consecutive focal planes showing the oral primordium of a protargol-stained late divider in longitudinal section.

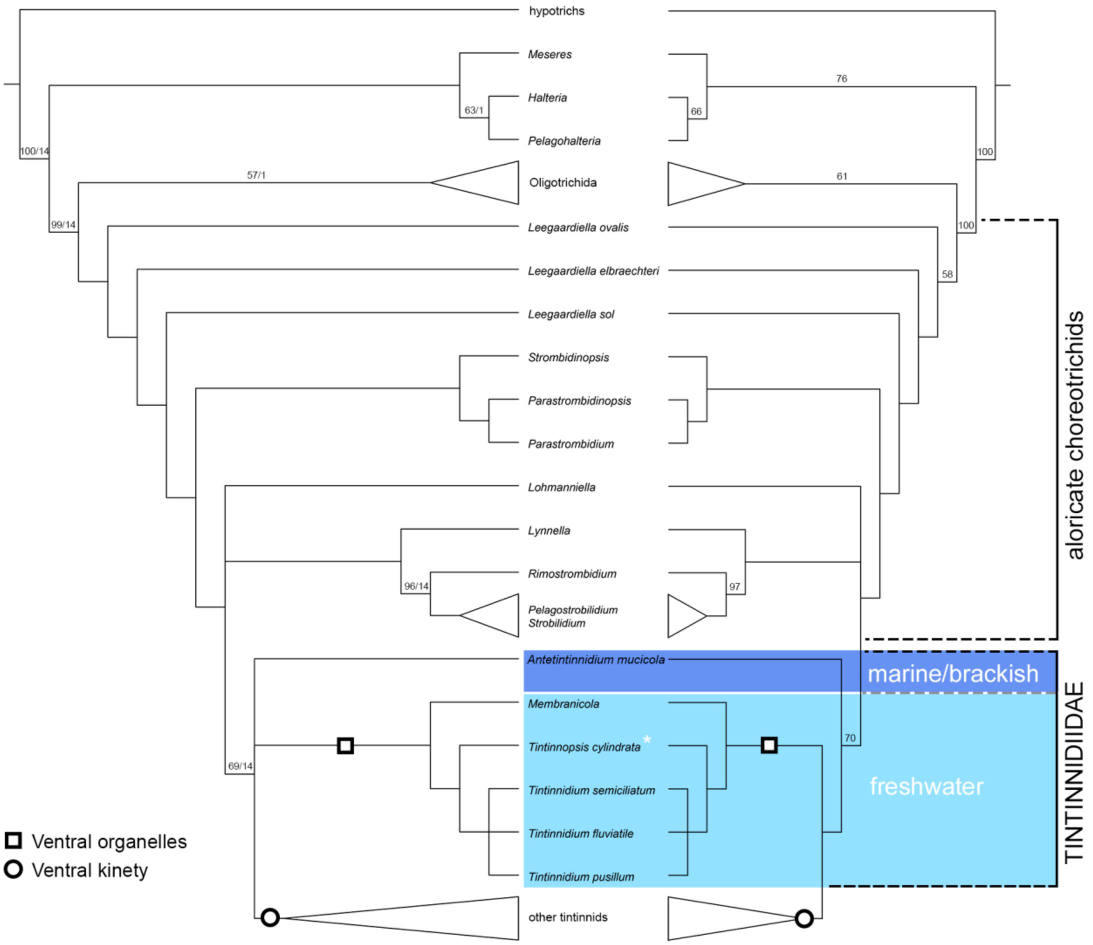

hypotrichs
Meseres
Halteria
Pelagohalteria
Oligotrichida
Leegaardiella ovalis
Leegaardiella elbraechteri
Leegaardiella sol
Strombidinopsis
Parastrombidinopsis
Parastrombidium
Lohmanniella
Lynnella
Rimostrombidium
Pelagostrobilidium
Strobilidium
Antetintinnidium mucicola
Membranicola
Tintinnopsis cylindrata *
Tintinnidium semiciliatum
Tintinnidium fluviatile
Tintinnidium pusillum
other tintinnids
63/1
100/14
57/1
99/14
96/14
69/14
76
66
100
61
100
58
97
70
aloricate choreotrichids
marine/brackish
freshwater
TINTINNIDIIDAE
Ventral organelles
Ventral kinety

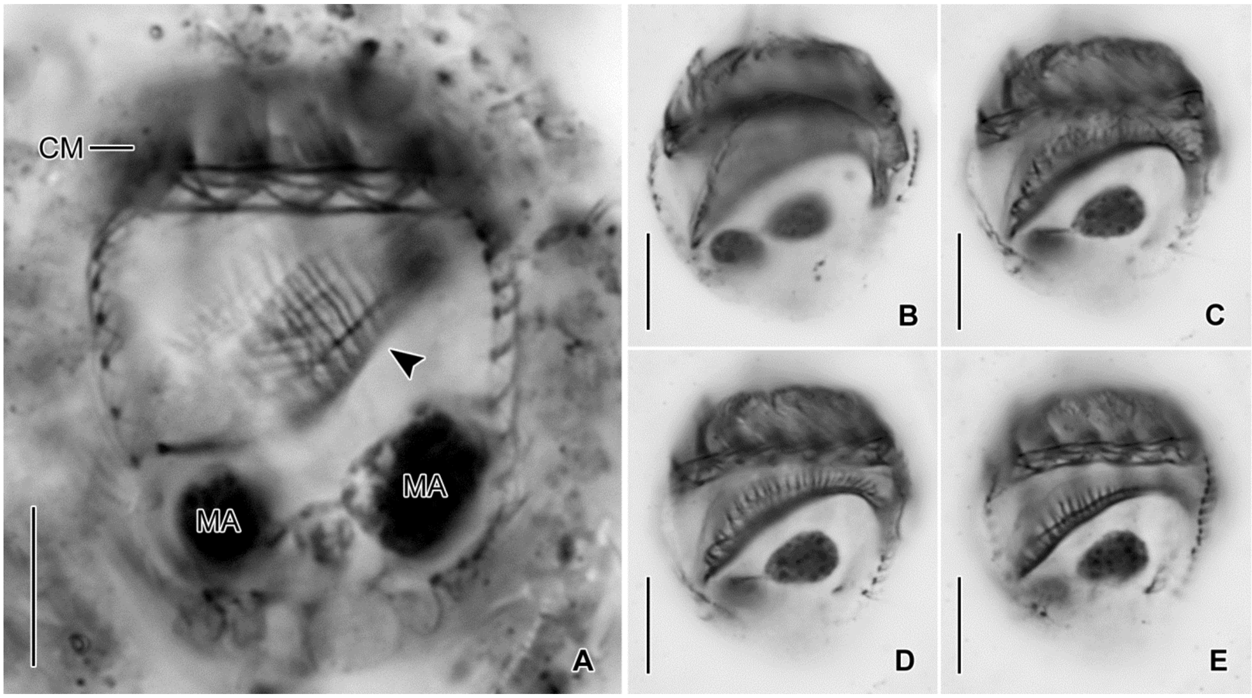
CM
MA
MA
A
B
C
D
E

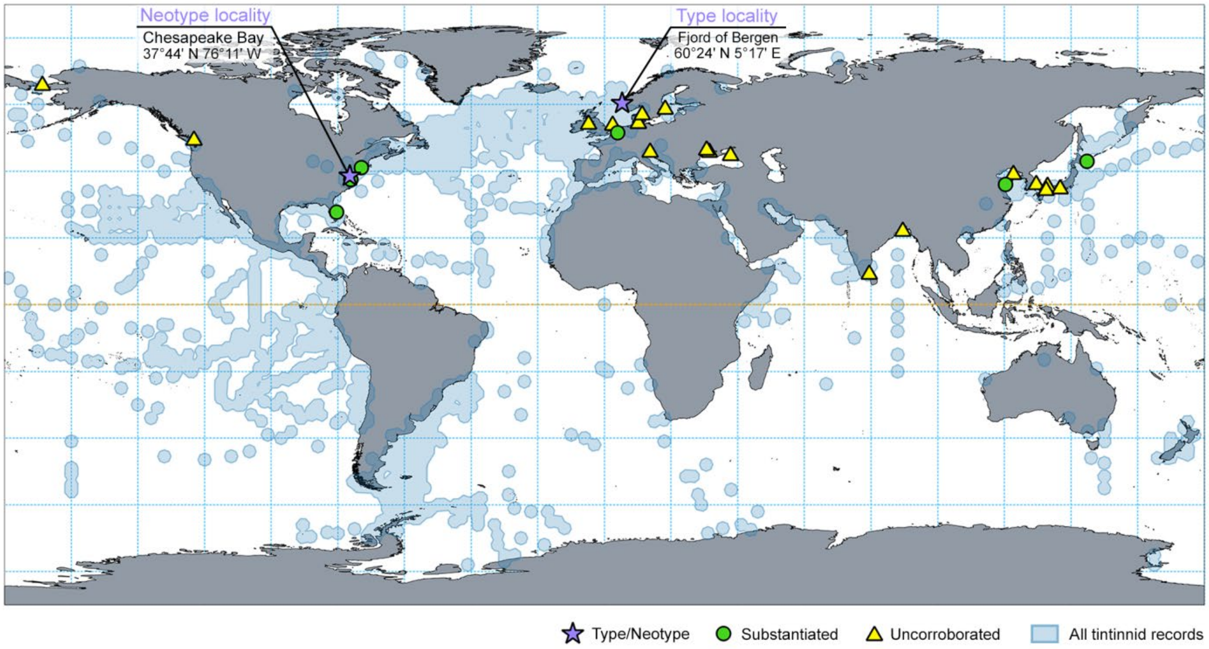
Neotype locality
Chesapeake Bay
37°44' N 76°11' W
Type locality
Fjord of Bergen
60°24' N 5°17' E
Type/Neotype
Substantiated
Uncorroborated
All tintinnid records

Table S1. Records of *Antetintinnidium mucicola* nov. gen., nov. comb. Categories (CAT): (1) From original type and neotype localities, (2) substantiated records, and (3) uncorroborated records

CAT	Reference	Oceanic region	Sampling location
1	Claparède and Lachmann (1858)	North Sea	Fjord of Bergen, Norway
1	This study	North Atlantic	Chesapeake Bay, USA
2	Brownlee (1977)[a]	North Atlantic	Delaware, USA
2	Dolan (1991)	North Atlantic	Chesapeake Bay, USA
2	Hada (1937)	Pacific Ocean	Akkeshi Bay, Japan
2	Santoferrara et al. (2013)	North Atlantic	Long Island Sound, USA
2	Strüder-Kypke and Lynn (2003)[b]	North Atlantic	Harbor Branch, USA
2	Tempelman and Agatha (1997)	North Sea	Oosterschelde, Netherlands
2	This study	North Sea	Wilhelmshaven, Germany
2	Zhang et al. (2017)	Yellow Sea	Qingdao, China
3	Anandakumar and Thajuddin (2013)	Indian Ocean	Gulf of Mannar, India
3	Biswas et al. (2013)	Indian Ocean	Ganges River Estuary, India
3	Blackbourn et al. (1973)	North Pacific	Vancouver, Canada
3	Dolgopolskaia (1940)	Black Sea	Karadag, Ukraine
3	Godhantaraman and Uye (2003)	Sea of Japan	Shinji, Ohashi River, Japan
3	Graziano (1989)	North Sea	Port Erin Bay, Isle of Man
3	Hansen-Ostenfeld (1916)	North Sea	Skagerak, Kattegat, Denmark
3	Hofker (1931)	North Sea	Scheveningen, Netherlands
3	Johansson et al. (2004)	Baltic Sea	Station BY31
3	Kim et al. (2007)	Sea of Japan	Kuryongpo, Korea
3	Kurilov (2003)	Black Sea	Lake Sukhoy Liman, Ukraine
3	Laackmann (1908)	Baltic Sea	Kiel Bight, Germany
3	Lindley (1975)	North Sea	Continuous plankton recorder
3	Lohmann (1908)	Baltic Sea	Kiel Bight, Germany
3	Matsuno et al. (2014)	Arctic Ocean	Chukchi Sea, Bering Strait
3	Monti et al. (2012)	Adriatic Sea	Trieste, Italy
3	Nagano and Uye (2002)	Pacific Ocean	Kure Port, Inland Sea of Japan
3	Nagano et al. (2001)	Pacific Ocean	Ise Bay, Japan
3	Schwarz (1959)	Baltic Sea	Kiel Bight, Germany
3	Selifonova (2001)	Black Sea	Bay of Novorossiisk, Russia
3	Uye et al. (2000)	Pacific Ocean	Ise Bay, Japan
3	Yu et al. (2013)	Yellow Sea	Zhangzi Island, China

[a]Unpublished Master Thesis.

[b]The authors provide a micrograph of a densely agglutinated lorica not clearly showing the lorica opening. Although verification of species identification is thus difficult, the SSU rDNA sequence is almost identical to the other sequences of *T. mucicola* specimens, indicating conspecificity.

SUPPLEMENTARY LITERATURE CITED

Anandakumar, N. & Thajuddin, N. 2013. Physico-chemical properties, seasonal variations in species composition and abundance of micro zooplankton in the Gulf of Mannar, India. *Indian J. Mar. Sci.,* 42:383-389.

Biswas, S. N., Gadhantaraman, N., Rakshit, D. & Sarkar, S. K. 2013. Community composition, abundance, biomass and production rates of tintinnids (Ciliata: Protozoa) in the coastal regions of Sundarban Mangrove wetland, India. *Indian J. Mar. Sci.,* 42:163-173.

Blackbourn, D. J., Taylor, F. J. R. & Blackbourn, J. 1973. Foreign organelle retention by ciliates. *J. Protozool.,* 20:286-288.

Brownlee, D. C. 1977. The significance of cytological characteristics as revealed by protargol silver staining in evaluating the systematics of the ciliate suborder Tintinnina. Master Thesis. University of Maryland, USA. ix + 146 p.

Claparède, É. & Lachmann, J. 1858. Études sur les infusoires et les rhizopodes. *Mém. Inst. natn. génev.,* 5 (year 1857):1-260 + Plates I-XIII.

Dolan, J. R. 1991. Guilds of ciliate microzooplankton in the Chesapeake Bay. *Estuar. coast. Shelf Sci.,* 33:137-152.

Dolan, J. R., Pierce, R. W. & Bachy, C. 2014. *Cyttarocylis ampulla*, a polymorphic tintinnid ciliate of the marine plankton. *Protist,* 165:66-80.

Dolgopolskaia, M. A. 1940. Das Zooplankton des Schwarzen Meeres in der Nähe von Karadag. *Trav. Stn biol. Karadagh,* 6:57-111 + Table.

Foissner, W., Berger, H. & Schaumburg, J. 1999. Identification and Ecology of Limnetic Plankton Ciliates. Informationsberichte des Bayer. Landesamtes für Wasserwirtschaft 3/99. Bayer. Landesamt für Wasserwirtschaft, Munich. p. 793.

Godhantaraman, N. & Uye, S. 2003. Geographical and seasonal variations in taxonomic composition, abundance and biomass of microzooplankton across a brackish-water lagoonal system of Japan. *J. Plankton Res.,* 25:465-482.

Graziano, C. 1989. On the ecology of tintinnids (Ciliophora: Oligotrichida) in the north Irish Sea. *Estuar. coast. Shelf Sci.,* 29:233-245.

Hada, Y. 1937. The fauna of Akkeshi Bay IV. The pelagic Ciliata. *J. Fac. Sci. Hokkaido Univ. (Zool.),* 5:143-216.

Hansen-Ostenfeld, C. 1916. De danske Farvandes Plankton i Aarene 1898-1901. Phytoplankton og Protozoer. 2. Protozoer; Organismer med usikker Stilling; Parasiter i Phytoplanktonter. *K. danske Vidensk. Selsk. Skr. (ser. 8),* 2:365-451.

Hofker, J. 1931. Studien über Tintinnoidea. *Arch. Protistenk.,* 75:315-402.

Johansson, M., Gorokhova, E. & Larsson, U. 2004. Annual variability in ciliate community structure, potential prey and predators in the open northern Baltic Sea proper. *J. Plankton Res.,* 26:67-80.

Kim, Y.-O., Chae, J., Hong, J.-S. & Jang, P.-G. 2007. Comparing the distribution of ciliate plankton in inner and outer areas of a harbor divided by an artificial breakwater. *Mar. Environ. Res.,* 64:38-53.

Kurilov, A. V. 2003. Features of planktonic ciliates assemblages in the Black Sea lagoon and estuaries of various types. *Ekol. Morya,* 64:7-12.

Laackmann, H. 1908. Ungeschlechtliche und geschlechtliche Fortpflanzung der Tintinnen. *Wiss. Meeresunters., Abt. Kiel,* 10:13-38 + Plates I-III.

Lindley, J. A. 1975. Continuous plankton records: a plankton atlas of the North Atlantic and North Sea: Supplement 3 - Tintinnida (Protozoa, Ciliophora) in 1965. *Bull. mar. Ecol.,* 8:201-213 + Plates XXVIII-XL.

Lohmann, H. 1908. Untersuchungen zur Feststellung des vollständigen Gehaltes des Meeres an Plankton. *Wiss. Meeresunters., Abt. Kiel,* 10:128-370 + Plates IX-XVII + Tables A, B.

Matsuno, K., Ichinomiya, M., Yamaguchi, A., Imai, I. & Kikuchi, T. 2014. Horizontal distribution of microprotist community structure in the western Arctic Ocean during late summer and early fall of 2010. *Polar Biol.,* 37:1185-1195.

Monti, M., Minocci, M., Milani, L. & Fonda Umani, S. 2012. Seasonal and interannual dynamics of microzooplankton abundances in the Gulf of Trieste (Northern Adriatic Sea, Italy). *Estuar. coast. Shelf Sci.,* 115:149-157.

Nagano, N. & Uye, S. 2002. Seasonal variations in abundance, biomass, in situ growth rate and production of tintinnid ciliates in Kure Port, the Inland Sea of Japan. *Bull. Soc. Sea Water Sci. Jpn,* 56:142-149.

Nagano, N., Iwatsuki, Y., Okazaki, Y. & Nakata, H. 2001. Feeding strategy of Japanese sand lance larvae in relation to ciliated protozoa in the vicinity of a thermohaline front. *J. Oceanogr.,* 57:155-163.

Santoferrara, L. F., McManus, G. B. & Alder, V. A. 2013. Utility of genetic markers and morphology for species discrimination within the order Tintinnida (Ciliophora, Spirotrichea). *Protist,* 164:24-36.

Schwarz, S. 1959. Vergleichende Studien an tierischem Netz- und Vollplankton aus Brackwassergebieten der Ostsee. *Z. Fisch.,* 8:351-370.

Selifonova, Z. P. 2001. Heterotrophic nano- and microplankton under conditions of anthropogenic eutrophication of the Bay of Novorossiisk. *Russ. J. Ecol.,* 32:266-271.

Strüder-Kypke, M. C. & Lynn, D. H. 2003. Sequence analyses of the small subunit rRNA gene confirm the paraphyly of oligotrich ciliates sensu lato and support the monophyly of the subclasses Oligotrichia and Choreotrichia (Ciliophora, Spirotrichea). *J. Zool., Lond.,* 260:87-97.

Tempelman, D. & Agatha, S. 1997. Biomonitoring van microzoöplankton in de Nederlandse zoute wateren 1996. *TRIPOS for Rijkswaterstaat, Rijksinstituut voor Kust en Zee,* 97.T0017-1b:i-ii + 1-77.

Uye, S., Nagano, N. & Shimazu, T. 2000. Abundance, biomass, production and trophic roles of micro- and net-zooplankton in Ise Bay, Central Japan, in winter. *J. Oceanogr.,* 56:389-398.

Yu, Y., Zhang, W., Wang, S. & Xiao, T. 2013. Abundance and biomass of planktonic ciliates in the sea area around Zhangzi Island, Northern Yellow Sea. *Acta Ecol. Sin.,* 33:45-51.

Zhang, Q., Agatha, S., Zhang, W., Dong, J., Yu, Y., Jiao, N. & Gong, J. 2017. Three rDNA loci-based phylogenies of tintinnid ciliates (Ciliophora, Spirotrichea, Choreotrichida). *J. Eukaryot. Microbiol.,* 64:226-241.

Table S2. List of 18S rDNA sequences used for calculation of maximum likelihood tree

**Isolate name**	**Accession No.**
Amphorellopsis acuta	JX101847
Amphorellopsis quinquealata	JQ924059
Amphorides amphora	JX101849
Amphorides minor	KY290324
Amphorides quadrilineata	JQ408184
Antestrombidium agathae	JX310365
Apostrombidium parakielum	JX025560
Ascampbelliella acuta	KY290315
Climacocylis scalaria	JQ408210
Climacocylis scalaroides	KY290330
Codonaria cistellula	JQ408167
Codonaria sp.	JQ408172
Codonella apicata	EU399531
Codonella aspera	JQ408166
Codonella cratera	DQ487193
Codonellopsis americana	AY143571
Codonellopsis gaussi	JQ924053
Codonellopsis morchella	JQ408173
Codonellopsis nipponica	FJ196072
Codonellopsis orthoceras	JQ408180
Coxliella sp.	JX101851
Cymatocylis calyciformis	JQ924046
Cymatocylis convallaria	JQ924050
Cymatocylis drygalskii	JQ924052
Cyrtostrombidium longisomum	KJ534582
Cyrtostrombidium longisomum	KJ609053
Cyrtostrombidium paralongisomum	KJ534583
Cyttarocylis acutiformis	KY290316
Dadayiella ganymedes[a]	JX101852
Dartintinnus alderae	MF039886
Dictyocysta elegans	KY290318
Dictyocysta lepida	KT792929
Dictyocysta reticulata	EU399532
Epiplocylis undella	KY290319
Epiplocyloides ralumensis	JX101854
Eutintinnus apertus	JQ408195
Eutintinnus fraknoi	EU399534
Eutintinnus lususundae	JX101857
Eutintinnus medius	KY290320
Eutintinnus pectinis	AF399170
Eutintinnus pectinis	JN831766
Eutintinnus perminutus	KT792926
Eutintinnus sp.	AY143569

Eutintinnus sp.	JN831767
Eutintinnus stramentus	JX101859
Eutintinnus tenuis	JN871721
Eutintinnus tubulosus	JQ408187
Eutintinnus tubulosus	JX101855
Favella adriatica	JQ408215
Favella campanula	FJ422984
Favella campanula	JX101861
Favella ehrenbergii	GU574769
Favella markusovszkyi	JN871725
Favella panamensis	AY143572
Halteria grandinella	AF508759
Helicostomella subulata Cluster I	JN831780
Helicostomella subulata Cluster II	JN831781
Helicostomella subulata Cluster III	JN831784
Laackmanniella prolongata	JQ924056
Laboea strobila	KY290331
Leegaardiella sp.	KY290313
Leprotintinnus nordqvisti	KU715761
Limnostrombidium viride	KU525754
Lynnella semiglobulosa	FJ876965
Metacylis angulata	KY290322
Metacylis pithos	JX101862
Metacylis tropica	KP883283
Novistrombidium apsheronicum	FJ876958
Novistrombidium orientale	FJ422988
Novistrombidium sinicum	KU525744
Novistrombidium testaceum	AJ488910
Novistrombidium testaceum	FJ377547
Omegastrombidium elegans	KU525750
Oxytricha longa	AF508763
Parafavella parumdentata	KY290328
Parallelostrombidium conicum	JN712657
Parallelostrombidium ellipticum	KJ704987
Parallelostrombidium obesum	FJ422991
Parallelostrombidium paralatum	KU525748
Parastrombidinopsis minima	DQ393786
Parastrombidinopsis shimi	AJ786648
Parundella aculeata	KY290327
Pelagostrobilidium minutum	FJ876959
Pelagostrobilidium neptuni	AY541683
Pelagostrobilidium paraepacrum	FJ876963
Pelagostrobilidium sp.	KY290314
Petalotricha ampulla[b]	KY290317
Protorhabdonella curta	JX101863
Protorhabdonella simplex	KY290323

Pseudotontonia simplicidens	FJ422993
Pseudotontonia sp.	KY290332
Ptychocylis minor	KY290321
Rhabdonella elegans	JQ408175
Rhabdonella hebe	AY143566
Rhabdonella poculum	JX101864
Rhabdonella spiralis	KT792932
Rhizodomus tagatzi	JQ392572
Rimostrombidium lacustre (reported as *R. lacustris*)	DQ986131
Rimostrombidium veniliae	FJ876964
Salpingacantha undata	KY290325
Salpingacantha unguiculata	KY290326
Salpingella acuminata	EU399536
Salpingella acuminata	JQ408155
Schmidingerella arcuata	JQ837815
Schmidingerella quequenensis	KU715765
Schmidingerella taraikaensis	FJ196073
Sinistrostrombidium cupiformum	JX310366
Spirostrombidium agathae	KU525745
Spirostrombidium apourceolare	KU525746
Spirostrombidium schizostomum	KM222098
Spirostrombidium subtropicum	JN712658
Spirotontonia grandis	KU525755
Spirotontonia taiwanica	FJ715634
Spirotontonia turbinata	FJ422994
Steenstrupiella steenstrupii	KT792924
Stenosemella nivalis	FJ196074
Stenosemella pacifica	JN831787
Stenosemella sp.	KU715763
Stenosemella steini	KT792927
Stenosemella ventricosa	JQ408170
Stenosemella ventricosa	KU715764
Stenosemella ventricosa	EU399538
Strobilidium caudatum	AY143573
Strombidinopsis acuminata	FJ790209
Strombidinopsis batos	FJ881862
Strombidinopsis jeokjo	AJ628250
Strombidinopsis sinicum	KR263893
Strombidinopsis sp.	JQ028734
Strombidium apolatum	DQ662848
Strombidium basimorphum	FJ480419
Strombidium biarmatum	AY541684
Strombidium capitatum	KP260510
Strombidium caudispina	KP260513
Strombidium cf. *parastylifer*	KU525751

Strombidium chlorophilum	KM084726
Strombidium conicum	FJ422992
Strombidium crassulum	HM140389
Strombidium cuneiforme	KP260512
Strombidium guangdongense	KJ609049
Strombidium inclinatum	AJ488911
Strombidium oculatum	KM084727
Strombidium paracalkinsi	KJ737432
Strombidium paracapitatum	KP260511
Strombidium pseudostylifer	KM084728
Strombidium rassoulzadegani	AY257125
Strombidium stylifer	JX012185
Strombidium sulcatum	FJ377546
Strombidium triquetrum	KJ609052
Strombidium tropicum	KJ609050
Stylicauda platensis	JN831832
Stylonychia lemnae	AF508773
Tintinnidium balechi	JN831797; Santoferrara et al. (2013)
Tintinnidium balechi	JN831796*; Santoferrara et al. (2013)
Tintinnidium balechi	JN831795*; Santoferrara et al. (2013)
Tintinnidium cf. *primitivum*	KU715766*; Zhang et al. (2016)
Tintinnidium fluviatile	JQ408163; Bachy et al. (2012)
Tintinnidium mucicola	JN831798*; Santoferrara et al. (2013)
Tintinnidium mucicola	JN831799; Santoferrara et al. (2013)
Tintinnidium mucicola	JN831800*; Santoferrara et al. (2013)
Tintinnidium mucicola	KU715767*; Zhang et al. (2016)
Tintinnidium mucicola	AY143563*; Strüder-Kypke and Lynn (2003)
Tintinnidium pusillum[e]	DQ487200; Duff et al. (2008)
Tintinnidium sp.	JN831803*; Santoferrara et al. (2013)
Tintinnidium sp. 1	JN831801; Santoferrara et al. (2013)
Tintinnidium sp. 2	JN831802; Santoferrara et al. (2013)
Tintinnidium sp. 3	JN831804; Santoferrara et al. (2013)
Tintinnopsis acuminata	JN831839
Tintinnopsis baltica	JN831805
Tintinnopsis beroidea	EF123709
Tintinnopsis bütschlii (reported as *T. buetschlii*)	JN831808
Tintinnopsis cf. *cylindrica*	MG603624
Tintinnopsis cylindrica	JN831811
Tintinnopsis cylindrica	JQ408206
Tintinnopsis dadayi	AY143562
Tintinnopsis everta	MG461220
Tintinnopsis fimbriata	AY143560
Tintinnopsis kiangsuensis[d]	JN831849
Tintinnopsis lacustris[c]	JQ408161
Tintinnopsis lata	KM982810

Tintinnopsis levigata	KM982811
Tintinnopsis lobiancoi	JN831813
Tintinnopsis lohmanni	FJ196076
Tintinnopsis major	JN831815
Tintinnopsis nana	JN831821
Tintinnopsis parva	JN831823
Tintinnopsis parvula	JN831825
Tintinnopsis pseudocylindrica	JN831853
Tintinnopsis radix	EU399540
Tintinnopsis rapa	JN831834
Tintinnopsis rara	JQ408200
Tintinnopsis subacuta	JN871724
Tintinnopsis tenuis	JN831848
Tintinnopsis tocantinensis	JN831835
Tintinnopsis tubulosoides	AF399108
Tintinnopsis turbinata	JN831846
Tintinnopsis urnula	JN831852
Tintinnopsis uruguayensis	JN831838
Tintinnopsis ventricosoides	KU715776
Undella claparedei	JQ408164
Undella hyalina	JQ408207
Undella marsupialis	JQ408214
Undella subcaudata	KT792931
Varistrombidium kielum	KJ609051
Williophrya maedai	FJ876966
Xystonella acus	KY290329
Xystonella longicauda	KT792933
**Environmental sequences**	**Accession No.**
Uncultured alveolate	EU162620*
Uncultured alveolate	HM135052*
Uncultured alveolate	FN690031*
Uncultured alveolate	FN690032*
Uncultured eukaryote	KJ925176*
Uncultured eukaryote	KJ925186*
Uncultured eukaryote	KJ925311*
Uncultured eukaryote	KJ925333*
Uncultured eukaryote	KJ925413*
Uncultured eukaryote	KJ925310*
Uncultured eukaryote	KJ925470*
Uncultured tintinnid	JX567482*

*Second dataset (n = 20) comprises environmental sequences and sequences of Tintinnidiidae which are not included in the first dataset and show less than 4% deviation from each other.

[a]*Dadayiella ganymedes* had probably been confused with *D. bulbosa*; [b]should possibly be affiliated with the genus *Cyttarocylis* (Dolan et al. 2014); [c]according to Foissner et al. (1999) a synonym of *Codonella cratera*; [d]possibly the senior synonym of *Stenosemella lacustris*;

[e]identification of sequenced specimen is not substantiated by an illustration and/or measurements.
